# Exome Sequencing of Phenotypic Extremes Identifies *CAV2* and *TMC6* as Interacting Modifiers of Chronic *Pseudomonas aeruginosa* Infection in Cystic Fibrosis

**DOI:** 10.1371/journal.pgen.1005273

**Published:** 2015-06-05

**Authors:** Mary J. Emond, Tin Louie, Julia Emerson, Jessica X. Chong, Rasika A. Mathias, Michael R. Knowles, Mark J. Rieder, Holly K. Tabor, Debbie A. Nickerson, Kathleen C. Barnes, NHLBI GO Exome Sequencing Project, Lung GO, Ronald L. Gibson, Michael J. Bamshad

**Affiliations:** 1 Department of Biostatistics, University of Washington, Seattle, Washington, United States of America; 2 Department of Pediatrics, University of Washington, Seattle, Washington, United States of America; 3 Center for Clinical and Translational Medicine, Seattle Children’s Research Institute Seattle, Washington, United States of America; 4 Department of Medicine, School of Medicine, Johns Hopkins University, Baltimore, Maryland, United States of America; 5 Cystic Fibrosis/Pulmonary Research and Treatment Center, University of North Carolina at Chapel Hill, Chapel Hill, North Carolina, United States of America; 6 Trueman-Katz Center for Pediatric Bioethics, Seattle Children’s Research Institute, Seattle, Washington, United States of America; 7 Department of Genome Sciences, University of Washington, Seattle, Washington, United States of America; 8 Division of Pulmonary Medicine, Seattle Children’s Hospital, Seattle, Washington, United States of America; 9 Division of Genetic Medicine, Seattle Children’s Hospital, Seattle, Washington, United States of America; The University of Queensland, AUSTRALIA

## Abstract

Discovery of rare or low frequency variants in exome or genome data that are associated with complex traits often will require use of very large sample sizes to achieve adequate statistical power. For a fixed sample size, sequencing of individuals sampled from the tails of a phenotype distribution (i.e., extreme phenotypes design) maximizes power and this approach was recently validated empirically with the discovery of variants in *DCTN4* that influence the natural history of *P*. *aeruginosa* airway infection in persons with cystic fibrosis (CF; MIM219700). The increasing availability of large exome/genome sequence datasets that serve as proxies for population-based controls affords the opportunity to test an alternative, potentially more powerful and generalizable strategy, in which the frequency of rare variants in a single extreme phenotypic group is compared to a control group (i.e., extreme phenotype vs. control population design). As proof-of-principle, we applied this approach to search for variants associated with risk for age-of-onset of chronic *P*. *aeruginosa* airway infection among individuals with CF and identified variants in *CAV2* and *TMC6* that were significantly associated with group status. These results were validated using a large, prospective, longitudinal CF cohort and confirmed a significant association of a variant in *CAV2* with increased age-of-onset of *P*. *aeruginosa* airway infection (hazard ratio = 0.48, 95% CI=[0.32, 0.88]) and variants in *TMC6* with diminished age-of-onset of *P*. *aeruginosa* airway infection (HR = 5.4, 95% CI=[2.2, 13.5]) A strong interaction between *CAV2* and *TMC6* variants was observed (HR=12.1, 95% CI=[3.8, 39]) for children with the deleterious *TMC6* variant and without the *CAV2* protective variant. Neither gene showed a significant association using an extreme phenotypes design, and conditions for which the power of an extreme phenotype vs. control population design was greater than that for the extreme phenotypes design were explored.

## Author Summary

Whole exome and whole genome sequencing provide the opportunity to test for associations between expressed traits and genetic variants that cannot be tested with chip technology, particularly variants that are too rare to be included on chips designed for genome-wide association analysis. We used exome sequencing to identify variants in *CAV2* and *TMC6* that modify the age-of-onset of chronic *Pseudomonas aeruginosa* infection among children with cystic fibrosis, and validated our findings in a large cohort of children with cystic fibrosis. For a fixed number of study participants, it is known that the extreme phenotypes design provides greater statistical power than a random sampling design. In the extreme phenotypes design, one compares the frequency of a given set of genetic variants in one extreme of age-of-onset (early onset) to that in the other extreme (late onset). Here, we employed an alternative design that compares genetic frequencies in exomes sampled from one extreme to that among exomes from a large set of controls. We show that this design confers substantially greater statistical power for discovery of *CAV2* and *TMC6* and provide general conditions under which this single extreme versus control design is more powerful than the extreme phenotypes design.

## Introduction

Cystic fibrosis (CF) (MIM219700) is a life shortening, monogenic condition caused by mutations in *cystic fibrosis transmembrane conductance regulator* (*CFTR*) [1]. CF is associated with dysfunction in multiple exocrine organs (e.g. pancreas, intestine, liver, lung), but the primary cause of morbidity and mortality is progressive obstructive lung disease associated with persistent endobronchial infection and neutrophilic inflammation [2,3]. Specifically, individuals with cystic fibrosis are at high risk for *Pseudomonas aeruginosa* (*P*. *aeruginosa*) airway infection, with a typical overall pattern of progression from first acquisition, to more frequent infections, to chronic infection and then to chronic mucoid *P*. *aeruginosa*. Eventually, the airways of ~80% of adult patients are chronically infected with *P*. *aeruginosa*.

*P*. *aeruginosa* airway infection in individuals with CF is associated with increased morbidity and reduced survival [4–6]. In a large registry-based study, any history of *P*. *aeruginosa* airway infection at or before age 2 years was associated with reduced lung function, increased frequency of pulmonary exacerbations and reduced survival [4–6]. Onset of mucoid *P*. *aeruginosa* is associated with a sharp decline in lung function [7,8] and even higher mortality [9]. Collectively, these observations support a causal relationship between frequent *P*. *aeruginosa* infection and morbidity and mortality in CF. Current CF treatment guidelines focus on early detection and attempted eradication of *P*. *aeruginosa* airway infection prior to establishment of chronic infection [10]. Aggressive acute and maintenance antibiotic treatment of *P*. *aeruginosa* infection in individuals with CF in the U.S. has coincided with a rise in the median predicted survival from 28 to 38 years [11].

The age-of-onset of chronic *P*. *aeruginosa* infection of the lungs in individuals with CF varies widely, and the heritability for age-of-onset of chronic/persistent *P*. *aeruginosa* infection of the airway, independent of *CFTR* genotype, has been estimated to be 0.85 (on a scale of 0 to 1) [12]. Identification of host genetic factors could help delineate sub-populations of individuals for more aggressive monitoring and treatment; identify new targets to facilitate development of therapeutics; and lead to a better understanding of the pathophysiology of *P*. *aeruginosa* infection in CF. Additionally, finding modifiers of *P*. *aeruginosa* airway infection in CF could provide a model for studies to find disease modifiers of other Mendelian conditions and perhaps for complex traits, as well. To this end, an intense search to find genetic modifiers of airway *P*. *aeruginosa* infection in CF has been underway over the past decade.

Variants in *MBL2* and *SCL9A3* have been associated with first acquisition of airway *P*. *aeruginosa* in children with CF [13], [14], and nominally significant associations between age-of-onset of chronic *P*. *aeruginosa* infection have been reported recently for other lectin pathway genes: *FNC1*, *FNC2* and *MASP3* [15]. More recently, we used exome sequencing and an extreme phenotypes design to discover two variants in *DCTN4* associated with early onset of chronic *P*. *aeruginosa* airway infection [16]. However, even collectively, these variants explain only a small fraction of the genetic variance of risk for *P*. *aeruginosa* airway infection, indicating that other genetic modifiers of *P*. *aeruginosa* airway infection remain to be found.

To find variants/pathways that influence risk of early chronic *P*. *aeruginosa* infection in individuals with cystic fibrosis, we used a study design in which a single phenotypic extreme is compared to a control population. In contrast, in an extreme phenotypes study design, frequencies of variants in the two opposite s of the same phenotype in a study population are directly compared. In the extreme phenotypes design, power depends both on the group sizes and on the difference in variant frequencies between extremes (i.e., the empirical effect size). However under certain genetic architectures, most or all of the effect size can be due to enrichment (or depletion) of casual variants in only one extreme relative to the entire study population. In such cases, comparison of the single enriched (or depleted) extreme to the entire study population will increase statistical power.

We sampled CF individuals from each of two extremes of phenotype, generated exome data for each sample, and then compared each extreme separately to a large control exome data set in order to discover genetic variants associated with the phenotypic expression in each CF sample. One extreme comprised 85 individuals with cystic fibrosis and extreme early onset chronic *P*. *aeruginosa* infection; the other comprised 65 individuals with extreme late onset *P*. *aeruginosa* (S1 Fig). Study individuals were drawn from the Early *Pseudomonas* Infection Control (EPIC) Observational Study and the Genetic Modifier Study (GMS; a member of the North American CF Gene Modifier Consortium [17],[18]). The large control data set included 3,239 individuals of European ancestry who were ascertained to study non-lung disease phenotypes as part of the NHLBI Exome Sequencing Project (ESP). In both the extreme early onset *P*. *aeruginosa* vs. control population and the extreme late onset *P*. *aeruginosa* vs. control population comparisons, we discovered a gene associated with time to chronic *P*. *aeruginosa* infection in individuals with CF. Specifically, we found a variant in *CAV2*, encoding caveolin-2, was associated with increased age-of-onset (i.e., protective) and variants in *TMC6*, encoding transmembrane-like channel 6 were associated with decreased age-of-onset (i.e., deleterious). Both of these results were subsequently replicated in a large cohort of independent samples from the Early Pseudomonas Infection Control (EPIC) [19] study cohort. Furthermore, we demonstrate that the statistical power to find both genes was greater under the single extreme vs. control population design than under the extreme phenotypes design. Together, these findings provide proof-of-principle that use of exome sequencing and a single extreme phenotype vs. control population design can identify genetic modifiers of Mendelian conditions and may be generalizable to the search for rare variants underlying complex, common diseases.

## Results

### *CAV2* is associated with extreme early onset *P*. *aeruginosa* airway infection

**Fig 1:**
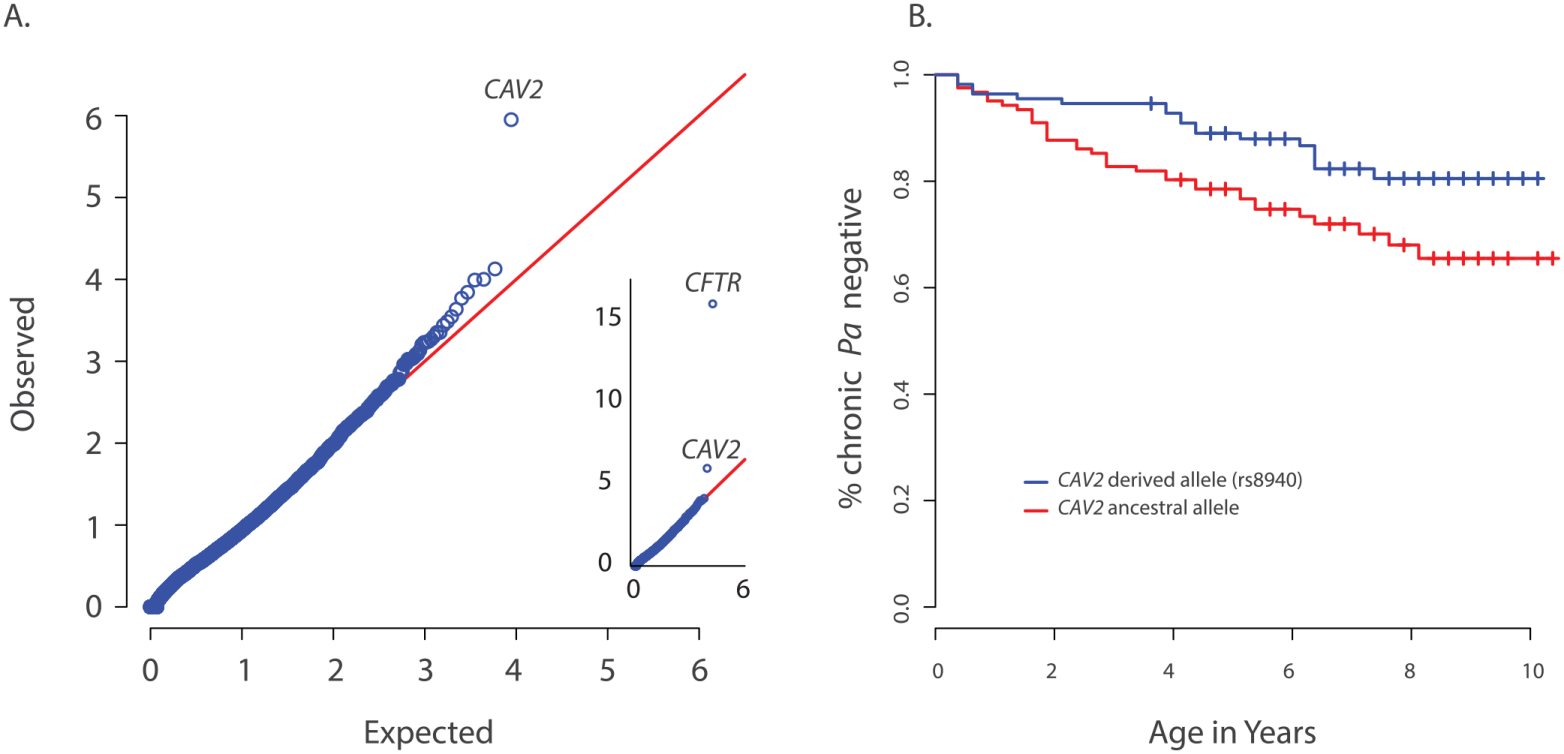
Primary results for *CAV2* from exome discovery and validation phases. (A) QQ-plot of p-values from the discovery analysis of exomes from 85 CF individuals with early onset chronic *P*. *aeruginosa* infection versus exomes from 3329 non-lung disease European American population controls. P-values are from the SKAT-O test with the small sample adjustment applied to non-synonymous variants grouped by gene to discover genes with differing distributions of these variants between groups. The most significant gene (highest point) is *CFTR* (p<1x10^-16^), followed by CAV2 (p = 1.1x10^-6^). 1(B) Kaplan Meier curves for age-of-onset of chronic *P*. *aeruginosa* infection among individuals in the validation cohort with (blue) and without (red) the *CAV2* rs8940 derived allele. Individuals in with at least one rs8940 variant had significantly later age-of-onset (HR = 0.53, p = 0.01, 95% CI = [0.32–0.88]).

A per-gene comparison between the extreme early onset chronic *P*. *aeruginosa* group (n = 85) and the control group (n = 3,239) using the optimal sequence kernel association test with small sample adjustment (aSKAT-O)[20] revealed significant differences between the frequencies of variants in two genes, *CFTR* (p<10^–16^) and *CAV2* (p = 1.1x10^-6^) (Fig 1A). Examination of the eight (nonsynonymous) variants included in the *CAV2* by-gene test showed that the aSKAT-O signal was likely due entirely to imbalance in frequency between groups for the single common variant (rs8940; p.(Q130E), MAF = 0.19 in European Americans (EA) per the NHLBI Exome Variant Server); the seven other variants had a cumulative MAF < 0.2%. This prediction was confirmed by a per-variant analysis in which the result for rs8940 replicated the by-gene result for *CAV2*, p = 6.7 x 10^–7^, for rs8940, which occurs at a highly conserved site (Genetic Evolutionary Rate Profiling (GERP) score = 5.9).

We attempted to validate the association between early age-of-onset of chronic *P*. *aeruginosa* and *CAV2* rs8940 by screening an additional 643 unrelated EPIC participants using the Infinium Human Exome BeadChip and manual genotyping of rs8940 among individuals for whom the exome chip genotyping was not performed (S1 Table). Association between the presence of at least one rs8940 minor allele and age-of-onset of chronic *P*. *aeruginosa* infection was tested using the Cox proportion hazards model stratified on age at entry to the EPIC study [16] The model was adjusted for *CFTR* risk group [21], number of bacterial cultures tested for each individual, presence of a *DCTN4* risk allele [16], age at enrollment and its interaction with rs8940. This model revealed a significant inverse association (p = 0.01; HR = 0.53, 95% CI = [0.32–0.88]) between age-of-onset of chronic *P*. *aeruginosa* infection and presence of at least one minor allele of rs8940 (Fig 1B and S2 Table). This effect was stronger among younger enrollees (p = 0.01 for rs8940 x enrollment age interaction.) Similar results were obtained after adjustment for ancestry using principal components (PCs) on the subset of 608 individuals with exome or genome-wide genotype chip data available (p = 0.0054; HR = 0.48, 95% CI = [0.29, 0.81]) (S2 Table).

*CAV2* is located ~1 Mb from *CFTR* and higher than expected frequencies of *CAV2* rs8940 alternate alleles were found in individuals with various *CFTR* mutations that cause CF, including *F508del-CFTR*, N1303K, G542X and W1282X (S3 Table), resulting in an association between *CFTR* mutation and CAV2 rs8940. Because age of acquisition and onset of chronic *P*. *aeruginosa* infection in CF might vary by *CFTR* mutation, and because *CFTR* mutations were associated with *CAV2* rs8940, there is potential for the association between rs8940 and time to chronic *P*. *aeruginosa* infection to be due to confounding. To remove any possible confounding by *CFTR* mutation, we repeated the Cox model analysis restricted to individuals homozygous for F508del*-CFTR* (n = 312); the results were essentially unchanged (p = 0.027; HR = 0.44, 95% CI = [0.21–0.91]). Even stronger results were obtained when the analysis of F508del*-CFTR* homozygotes was restricted to the individuals with self-declared European ancestry and adjusted for PCs (n = 286) (p = 0.0087; HR = 0.35, 95%CI = [0.16–0.77]) (S2 Table), but the difference in HR among the *F508del-CFTR* homozygous group and remaining individuals did not reach statistical significance (p = 0.13). These findings support a strong association between presence of the *CAV2* rs8940 alternate allele and protection against earlier and more severe *P*. *aeruginosa* infection, an association that is independent of the *CFTR* mutation.

### *TMC6* is associated with extreme late onset *P*. *aeruginosa* airway infection

**Fig 2:**
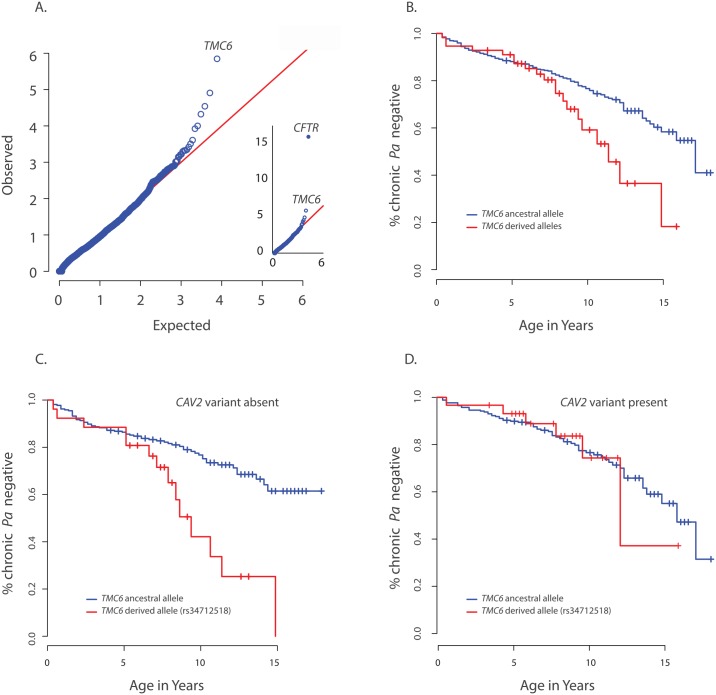
Primary results for *TMC6* from exome discovery and validation phases and its interaction with *CAV2*. (**A**) QQ-plot of p-values from the discovery analysis of exomes from 65 CF individuals with late onset chronic *P*. *aeruginosa* infection versus exomes from 3275 non-lung disease European American population control exomes. P-values are from the SKAT-O test with the small sample adjustment applied to non-synonymous variants grouped by gene to discover genes with differing distributions of these variants between groups. The most significant gene (highest point) is *CFTR* (p<1x10^-16^), followed by *TMC6* (p = 9.5x10^-7^). (**B**) Kaplan Meier curves for age-of-onset of chronic *P*. *aeruginosa* in individuals in validation cohort with cystic fibrosis with at least one *TMC6* rs3412518 derived allele versus those with ancestral alleles. Individuals with at least one *TMC6* rs3412518 derived allele had a higher risk for early onset chronic *P*. *aeruginosa* and this risk increased significantly over time (p = 0.01 for age interaction; HR = 5.2 at age 10, p = 0.00046, 95% CI = [2.1–43.0]). (**C**) Kaplan Meier curves for age-of-onset of chronic *P*. *aeruginosa* in individuals in validation cohort with cystic fibrosis showing *TMC6* risk groups among individuals *without* the *CAV2* rs8940 protective (derived) allele. Individuals with at least one *TMC6* rs34712518 derived allele had higher risk for early onset and this risk increased significantly over time (HR = 12.1 at age 10, p = 2.6x10^-5^, 95% CI = [3.8–38.8]). (**D**) Kaplan Meier curves for age-of-onset of chronic *P*. *aeruginosa* in individuals in validation cohort with cystic fibrosis showing *TMC6* risk groups among individuals with the *CAV2* rs8940 protective (derived) allele. There was no significant difference in risk between individuals with and without *TMC6* rs34712518 among these children with the *CAV2* protective allele (HR = 2.1, p = 0.41, 95% CI = [0.38–11.3]).

We next performed a per-gene comparison between the extreme late onset chronic *P*. *aeruginosa* airway infection extreme (n = 65) and the ESP control group above using aSKAT-O and found that the frequencies of variants in *CFTR* (p<1x10^-10^) and *TMC6* (p = 9.5x10^-7^) differed significantly between the two groups (Fig 2A). Examination of variants in *TMC6* in the late onset extreme vs. control group revealed a complex pattern of differences among the 36 non-synonymous variants contributing to the by-gene test (S4 Table). Specifically, the late onset group had fewer rare variants (cumulative observed MAF = 0.007 vs. 0.014), a higher observed MAF for two (rs12449858, rs2748427) of three common variants (rs34712518, rs12449858, rs2748427 with MAFs of 0.06, 0.09, and 0.19 per EVS, respectively) in *TMC6* and a lower derived allele frequency at rs3471258. Based on these observations, we next performed a validation analysis in which we collapsed all of the rare variants into one score (i.e., *TMC6* rare variant score) while the three common variants were analyzed individually. Thus we performed a total of four tests in the validation analysis using the Cox model as above for the *CAV2* validation.

The *TMC6* validation analysis was performed using data from 580 unrelated EPIC participants genotyped using the Illumina Exome chip. The presence of one or more derived alleles at rs34712518 was significantly associated with age-of-onset of chronic *P*. *aeruginosa* infection in the primary test of association (p = 0.012, HR = 1.8, 95% CI = [1.3–2.8], p< 0.05 after correction for the four tests). The Kaplan Meier plot comparing age-of-onset of chronic *P*. *aeruginosa* between *TMC6* rs34712518 variant groups showed a striking divergence in risk with progressing age (Fig 2B), indicating the hazard ratio is dependent on age (i.e. the hazards are not proportional over ages) and that an age-allele interaction should be fitted in the Cox model for a more accurate description of the effect. At age 7 years, the HR was 4.5 (p = 0.01, [1.4–12.6]), and at 10 years, the HR was 5.2 (p = 0.00046, [2.1–43.0]) (S5 Table). Variant rs12449858 was not significantly associated with age-of-onset of chronic *P*. *aeruginosa* (p = 0.12, HR = 0.7, 95% CI = [0.44, 1.1]), nor was the collapsed rare variant score (p = 0.10, HR = 0.38, 95% CI = [0.12–1.2]).

The derived allele of *TMC6* rs2748427 also was marginally associated with age-of-onset of chronic *P*. *aeruginosa* infection (p = 0.027, HR = 1.4 95% CI = [1.03–1.8], p = 0.08 after multiple-test correction). However, there was a strong association between the derived allele counts of rs3412518 and rs2748427 (Pearson correlation = 0.5; p<2x10^-16^), and individuals with a derived rs2748427 allele but not a derived rs34712518 allele showed no independent risk of chronic *P*. *aeruginosa* infection (HR = 1.18, p = 0.29, [0.86–1.6]). This suggests that the variant with a lower MAF, rs34712518 (MAF = 0.06; p.(G191D)) is associated with higher risk while the more common variant, rs2748427, is not independently associated with increased risk. The result was similar after adjusting for PCs and limiting the analysis to *F508del-CFTR* homozygotes (S5 Table). There were no significant interactions with enrollment age nor with chronological age in either case.

We tested for interactions between risk variants found in *DCTN4*, *CAV2*, and *TMC6*. No significant interaction was identified between *DCTN4* and either *CAV2* or *TMC6*. In contrast, we did find a significant interaction between the derived rs3412518 allele in *TMC6* and the rs8920 *CAV2* allele (p = 0.007). Specifically, by age 10, children with the derived *rs3412518* allele in *TMC6* without the protective *CAV2* allele were at much higher risk of chronic *P*. *aeruginosa* infection (p = 2.6x10^-5^, HR = 12.1, [3.8–38.8] (Fig 2C), while children with the derived rs3412518 allele in *TMC6* with the protective *CAV2* variant had no significant excess risk of chronic *P*. *aeruginosa* infection at this same age (p = 0.41, HR = 2.1, [0.38–11.3]) (Fig 2D and S5 Table). When the test for the *TMC6-CAV2* interaction was limited to the *F508del*-*CFTR* group, the test was again significant (p = 0.03) with an estimated HR = 29.1 at age 10 for *F508del-CFTR* homozygous children with both the *TMC6* and *CAV2* deleterious alleles (95% CI = [4.1, 204.8], n = 129, S3 Fig) compared to HR = 2.6 (95% CI = [0.3, 23.1], n = 144) for those with the TMC6 deleterious allele and a protective (derived) CAV2 allele.

### *CAV2* and *TMC6* are associated with lung function

**Fig 3:**
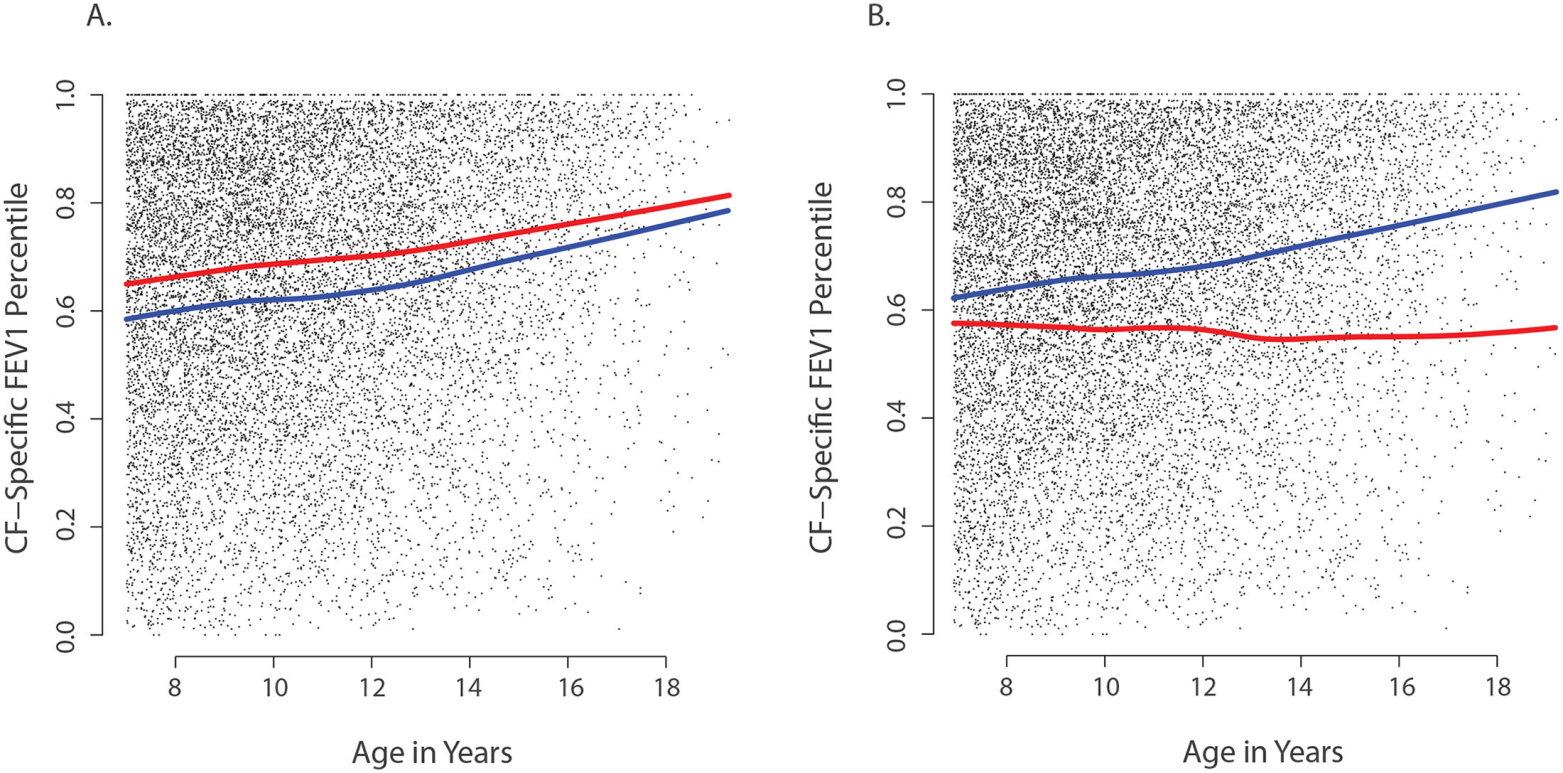
Association of *CAV2* and *TMC6* variants with lung function. Each small dot in each panel represents one CF-specific FEV1 percentile value in the data set (percentiles are age- and sex-adjusted); and each individual has an average of 3.5 percentile values per year in the data set. (**A**) Mean CF-specific FEV1 percentile by age for 289 EPIC validation individuals with (red) and 283 without at least one CAV2 rs8940 derived allele (blue). FEV1 was an average of 5.5 percentile points higher among individuals with the rs8940 derived allele (p = 0.001). (**B**) Mean CF-specific FEV1 percentile by age for 59 EPIC validation individuals with (red) and 513 without at least one TMC6 rs34712518 derived allele (blue). FEV1 was an average of 8.0 percentile points lower among individuals with the rs34712518 derived allele (p = 0.01). The apparent age interaction in the diagram (growing distance between lines) was not significant here, though power is limited by the lower numbers of observations at older ages.

Because acquisition of *P*. *aeruginosa* is associated with worse lung function over time, strong modifiers of *P*. *aeruginosa* infection may also be associated with lung function. Accordingly, we tested for association between *CAV2* rs8940 and *TMC6* rs34712518 cystic fibrosis-specific percentiles of forced expiratory volume at 1 second (FEV1). Among 572 children with lung function data beyond age 7 years, an age at which lung function studies are considered reliable, the presence of one or more *CAV2* rs8940 derived alleles was associated with a 5.5 percentile point increase in lung function (p = 0.001) (Fig 3A), while presence of one or more *TMC6* rs34712518 alleles was associated with an 8.0 percentile point decrease in CF-specific FEV1 (p = 0.01) (Fig 3B). Both of these effects were in the direction predicted based on their association with time to chronic *P*. *aeruginosa* infection. No interaction effect between variants was detected for the FEV1 outcome.

### Power of the single extreme phenotype vs. control population design

**Fig 4:**
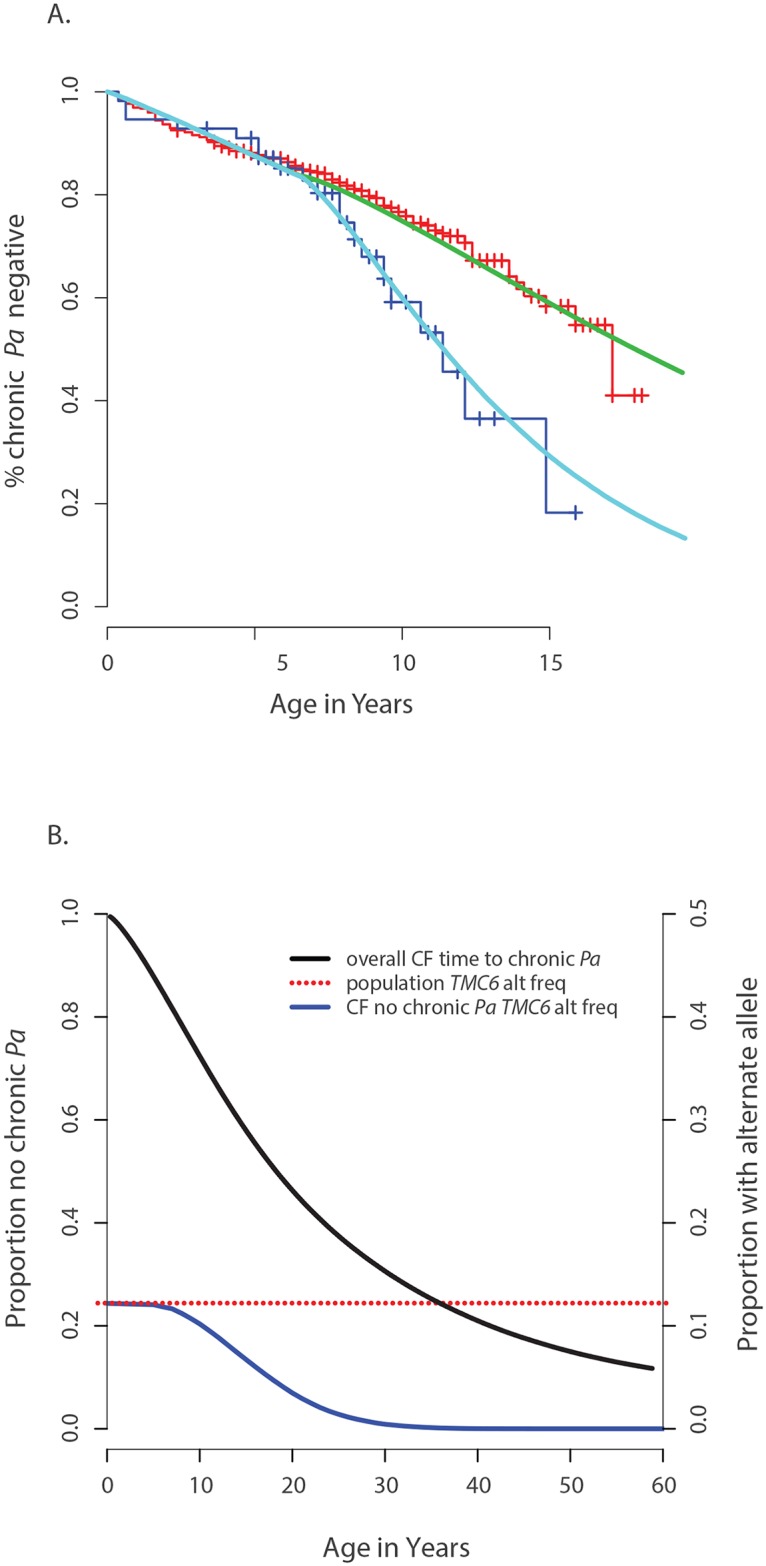
Genetic architecture of age-of-onset of chronic *P*. *aeruginosa* infection and TMC6 rs34712518. (**A**) Observed age of onset for children with at least one rs34712518 allele (blue) and without (red), compared to Weibull distribution models for age of onset among children with at least one rs34712518 allele (light blue) and without (green). (**B**) Blue: The probability of carrying at least one rs34712518 allele given age of onset is beyond age X (i.e. one does not have chronic *P*. *aeruginosa* infection by age X) based on the event probabilities for each carrier state shown in (A). The probability of being a carrier declines markedly between ages 6 and 25 among “survivors;” the more extreme (later) ages of onset have a growing difference in proportion of carriers compared to the general population (dotted red line), conferring greater power when included in the sample. Power provided by a single extreme vs. control design can be calculated from this plot, given the ages of the individuals in the single extreme.

We sought to understand to what extent discovery of these associations statistical power afforded by using the single extreme vs. control population design instead of an extreme phenotypes design. We performed a detailed power study for *TMC6*, using simulations based on the observed association between *TMC6* and age-of-onset of chronic *P*. *aeruginosa* airway infection in the validation data set. The results not only show explicit power gains but the methods also serve as a model for power calculations for other studies. Specifically, the distributions of event times for CF individuals with and without the rs3412518 alternate allele were well described by different Weibull distributions (Fig 4A). The Weibull distribution is widely used in time-to-event analysis because of its flexibility and interpretability [22]. We calculated the expected frequency of the *TMC6* rs3412518 alternate allele as a function of age among children who had not yet reached chronic *P*. *aeruginosa* airway infection by that age (Fig 4B). The results show a striking depletion of the rs3412518 alternate allele among CF individuals who were older and free of chronic *P*. *aeruginosa* airway infection. This is expected, intuitively, for a modestly rare allele that confers high risk: individuals carrying the alternate allele will succumb earlier to chronic *P*. *aeruginosa* airway infection and will be under-represented among individuals free of *P*. *aeruginosa* at older ages.

**Fig 5:**
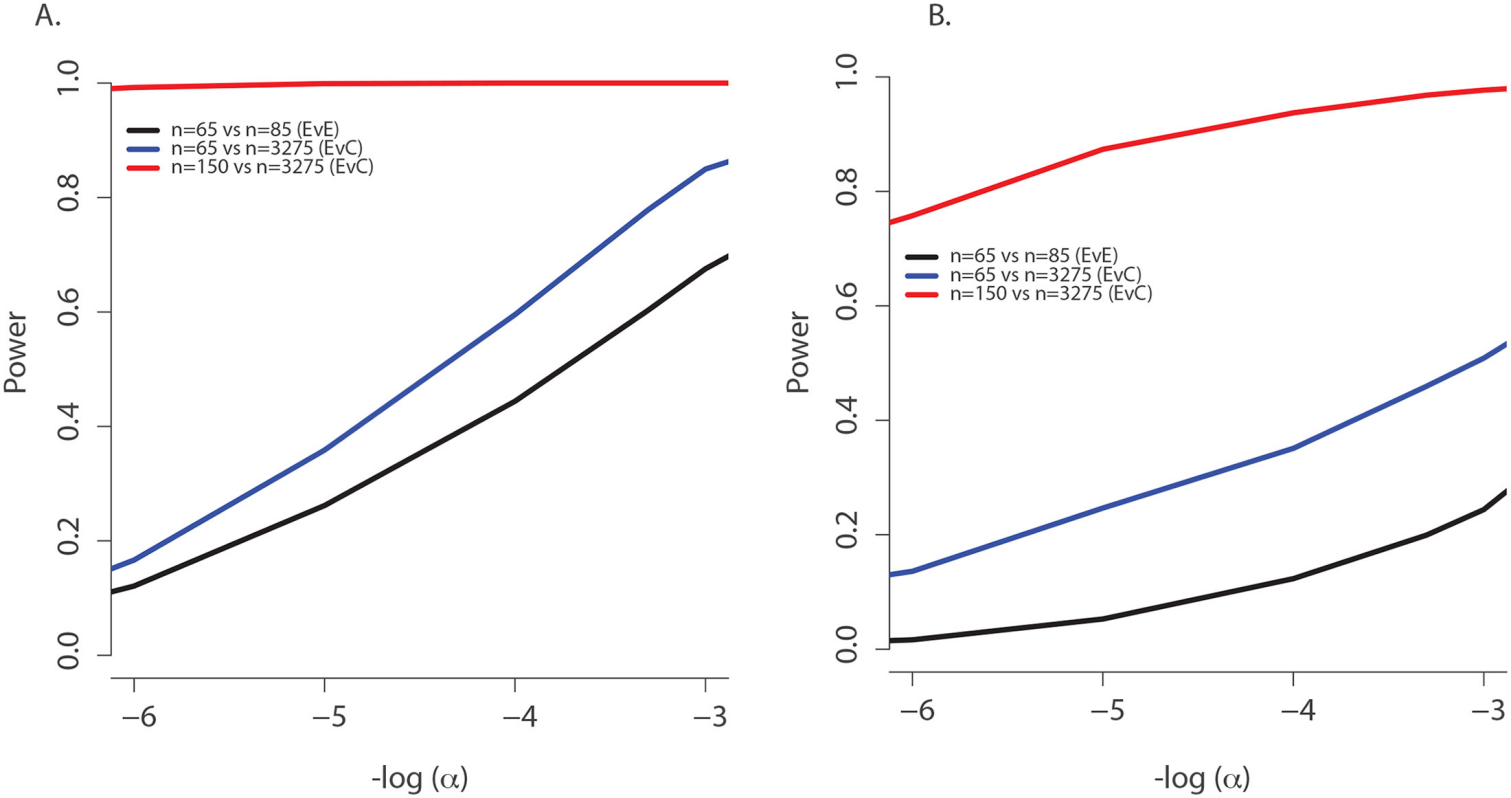
Power to discover *TMC6* with aSKAT-O for a test of size α under the actual ages of the individuals in the extreme samples in this study. (**A**) Power to detect a difference between groups at *TMC6 rs34712518* for three study designs based on the allele distributions for actual ages of individuals in the extremes in this study: extreme phenotypes (i.e. extreme versus extreme; black); the single extreme versus population control study reported here (blue); hypothetical single extreme versus control study if all exome resources had been devoted to the late onset extreme, which provides near 99% power to detect this variant (red). (**B**) Power to detect a difference between groups for *TMC6* by-gene assuming the observed allele frequencies in the validation set (for CF individuals) and in ESP controls. Study designs are the same as in (A). The power of the single extreme versus control design to detect *TMC6* at the exome-wide significance level of 2x10^-6^ is approximately 20% (blue), which is an order of magnitude higher than that of the extreme phenotypes design (black), while devoting all exomes to one extreme would provide nearly 80% power (red).

The probability of carrying a *TMC6* rs3412518 alternate allele can be calculated for children who became chronically infected with *P*. *aeruginosa* at a particular age to obtain the *TMC6* allele distributions among our extreme samples for the observed ages in each extreme group (using the same methods that were used to generate Fig 4B). New samples, conditional on the observed ages, were drawn from the Weibull-based distributions, and the power of aSKAT-O to detect a difference between groups was calculated for our sample sizes. The result was an approximately 40% increase in power across a range of test sizes for the single extreme phenotype vs. control design relative to the extreme phenotypes design for *TMC6* rs3412518 (Fig 5A). We also calculated the power based on a sample size of 150 (i.e., all available exomes originating from one extreme) in the late onset chronic *P*. *aeruginosa* airway infection extreme vs. population control and observed exceptional power for the single extreme vs. control design (99%) when all exomes were devoted to the single extreme (Fig 5A, red line). This is consistent with the increasing differences in the population MAF and the allele frequency in the late age-of-onset extreme (Fig 4B, red dotted line versus blue line, respectively.) In a separate power analysis, we drew samples from the observed distributions of variants over *TMC6* (S4 Table) to better represent a by-gene analysis. Power gains were even greater under this scenario for the single extreme vs. control design relative to the extreme phenotypes design (S5B Fig). Doubling the sample size of the extreme in a single extreme vs. control design provides greater power than the extreme phenotypes design even when the allele frequency of the causal variant in both extremes differs from the population frequency.

## Discussion

We used a single extreme phenotype vs. control population study design to discover variants in *TMC6* and *CAV2* associated with age-of-onset of chronic *P*. *aeruginosa* airway infection in individuals with CF. Examination of the variants within *CAV2* and *TMC6* allowed us to tailor our validation analysis to provide additional information on the role of specific variants that could not be uncovered in the by-gene discovery analysis. We found that *CAV2* rs8940 was associated with protection against chronic *P*. *aeruginosa* airway infection and *TMC6* rs34712518 was associated with earlier age-of-onset of chronic *P*. *aeruginosa* airway infection. Among individuals with both variants in the validation set, the protective effect of *CAV2* rs8940 nullified the deleterious effect of *TMC6* rs34712518 (i.e., a significant interaction was present rather than an additive effect). Consistent with these results, we also found a significant difference in lung function between children with and without *CAV2* rs8940 and *TMC6* rs34712518. Notably, the FEV effect size for *TMC6* rs34712518 is of the same magnitude as that for risk group 1 vs. risk group 2 *CFTR* genotype groups in this cohort (no functional CFTR versus some residual function, respectively, in risk groups 1 and 2 [21]). Together, these findings suggest that both *CAV2* and *TMC6* are strong modifiers of age-of-onset of chronic *P*. *aeruginosa* airway infection in persons with CF. Neither of these discoveries were possible using the extreme phenotypes (extreme vs. extreme) analysis with our samples, with p-values for both genes being greater than 0.001 under that design.

The observation that *CAV2* is a modifier of age-of-onset of chronic *P*. *aeruginosa* airway infection in individuals with CF is strongly supported by functional studies of caveolin-2 and its dimeric complement, caveolin-1. Caveolin-2 is one of a family of three structural proteins of caveolae, flask-shaped invaginations on the surface of lung epithelium and myeloid cells (Zaas 2009–1)[23]. Caveolins contribute to host defenses by regulating lipid-raft-mediated endocytosis and play a substantial role in the inflammatory response to infection and protein trafficking in the lung epithelium of individuals with CF [24,25]. In models of invasive infection, *P*. *aeruginosa* co-opts the host endocytotic machinery of lung epithelial cells where it replicates. In a variety of human cell lines, caveolin-2 forms a heterodimer with caveolin-1, which after infection with *P*. *aeruginosa*, co-localizes with CFTR and *P*. *aeruginosa* [26]. Knock-out of *CAV2* in murine epithelial cells prevents *P*. *aeruginosa* invasion [27]. Notably, *CAV2* is among the genes identified by GeneGO in conjunction with the Cystic Fibrosis Foundation in the MetaMiner-CF database and network tool as potential modifier genes in CF based on its role in endosome formation in lung epithelial cells [28]. PolyPhen2 classifies the CAV2 rs8940 variant as “probably damaging,” with a score of 0.997, while the Combined Annotation-Dependent Depletion (CADD) score puts this variant among the 2% of SNVs most likely to be pathogenic [29] (CADD-PHRED score = 17.1)(http://cadd.gs.washington.edu/download, V1.1).

The role of lung epithelial cell invasion in chronic *P*. *aeruginosa* airway infection in persons with CF remains unknown [30]. However, invasion of host epithelial cells at some point in the development of chronic *P*. *aeruginosa* airway infection is suggested by the presence of anti-*Pseudomonal* antibodies early in life among persons with CF infected with *P*. *aeruginosa* [31] and a spike in *anti-P*. *aeruginosa* antibody titers in persons with CF prior to diagnosis of chronic *P*. *aeruginosa* airway infection [7,32]. This suggests that variants in *CAV2* might reduce early *P*. *aeruginosa* invasion of lung epithelia, thereby reducing the risk of chronic *P*. *aeruginosa* airway infection. Alternatively, co-expression of caveolin-2 with *F508del-CFTR* in murine cell lines can rescue mutant CFTR expression on the cell surface [33]. Isoforms of caveolin-2 might rescue mis-folded CFTR proteins (e.g., *F508del-CFTR*) resulting in increased cell surface expression of mutant *CFTR* and improved lung defenses against *P*. *aeruginosa*. While the mechanism(s) by which *CAV2* rs8940 influences risk of chronic *P*. *aeruginosa* airway infection in persons with CF remains to be determined, our results are consistent with the known functional roles of caveolins in response to *P*. *aeruginosa* infection in CF models and highlight caveolin-2 as a potential therapeutic target to block *P*. *aeruginosa* invasion.

*TMC6* encodes a highly conserved, integral transmembrane protein that interacts with zinc transporter 1 to influence intracellular zinc concentrations. Mutations in *TMC6* underlie epidermodysplasia verruciformis, a condition in which persons are unusually sensitive to development of skin lesions caused by invasion human papilloma virus (HPV) [34]. One postulated mechanism is that variants in *TMC6* dysregulate HPV replication via an effect on AP-1 transcription [35]. AP-1 plays a cooperative role in the *P*. *aeruginosa*-dependent induction of IL-8, a key mediator in human bronchial epithelial cells and the lung pathology of persons with CF [36]. In addition, an AP-1 transcription factor binding site is located in the promoter of *DEFB1*, which encodes beta-defensin-2 [37], another important cytokine involved in protection against *P*. *aeruginosa* in persons with CF. Accordingly, variants in *TMC6* may influence the regulation of AP-1 and its downstream effectors, ultimately modifying the response to *P*. *aeruginosa*. Moreover, HPV gains entry to cells via both clathrin-dependent and caveolin-dependent endocytosis [38,39]. *TMC6* could affect HPV invasion at the point of caveolin-mediated endocytosis, functionally linking *TMC6* and *CAV2* and providing a basis for the observed interaction between *TMC6* and *CAV2* on risk of chronic *P*. *aeruginosa* airway infection. These results provide a compelling reason to pursue functional studies of the associated variants. However, it should be kept in mind that causal variants may in LD with the discovery variants.

We identified a strong interaction between risk variants in *CAV2* and *TMC6*. This finding highlights two considerations for studies aimed at identifying risk variants for complex traits. First, to increase the power of association studies for novel risk variants, it might be necessary to test for interactions with known genetic risk variants. This is feasible from a multiple-testing standpoint, given the relatively small numbers of known risk variants. Second, the possibility that a substantial portion of the “missing heritability” might be due to interactions among risk variants should emphasize recent attention to development and use of methods for identifying significant interactions in the face of multiple-testing in discovery analyses, a difficult but important problem.

We used a single extreme phenotype vs. control population design to gain power over an extreme phenotypes design to identify genes with differences in variant distributions between groups. Our power studies based on modeling of observed distributions for variants in *TMC6* revealed that use of this strategy resulted in substantial power gains over the extreme phenotypes design. The power gain for identifying the association between age-of-onset of chronic *P*. *aeruginosa* airway infection and *TMC6* variants derives from the observation that the late onset chronic *P*. *aeruginosa* airway extreme was depleted of *TMC6* risk variants, while the frequency of these risk variants in the extreme early onset chronic *P*. *aeruginosa* airway infection did not differ substantially from the control population. Indeed, since the *TMC6* risk variant has little effect until age 5 to 7 years, selecting children with age-of-onset before age 7 years did not enrich for this risk variant. That risk variants were depleted in the population of “survivors” (children with later age-of-onset in this setting) is an important consideration for studies to discover risk variants for childhood-onset conditions such as CF because the direct implication is that cohorts of adults can be underpowered to detect risk variants that affect the phenotype early in life.

The relative power of a single extreme phenotype vs. control population compared to an extreme phenotypes design will depend on the genetic architecture underlying the phenotype studied. In either design, selecting extreme individuals in at least one “case” arm of the study will result in enrichment of causal variants in that arm relative to both population controls and to the opposite extreme, a fact that can be demonstrated by Bayes Theorem, but the degree of relative enrichment (effect size) might differ between designs. Given N samples in each extreme, the extreme phenotypes design will usually be more powerful than a single extreme phenotype vs. control for situations in which both extremes exhibit differences in variant distributions relative to population controls, regardless of how large the sample size is for the control population. This is due to the diminishing “rate of return” in power as the control population sample size increases while the “case” sample size stays fixed at N. On the other hand, we found no realistic situation in which an extreme phenotypes design with N samples per arm had better power than a 2N single extreme vs. control population design. The implication is that investigators with access to control exomes/genomes might elect to allocate resources entirely to one phenotypic extreme so as to maximize power. This is fortuitous because only one extreme can be defined well or is of greater biomedical interest for many conditions (e.g., high blood pressure, risk of early stroke, etc.). Consequently, we think the single extreme phenotype vs. control population design is likely to be a desirable approach, particularly for adult-onset common diseases.

Generating exome/genome data from large control populations is expensive and as additional exomes/genomes become publically available, investigators will increasingly rely on public repositories of sequence data (e.g., dbGaP) for discovery studies. Such studies should always be validated with appropriate independent samples, but attention should still be given to avoiding false positives at the discovery stage. Our extreme and control exomes were sequenced contemporaneously at the same lab, but factors such as differences in targets, sequencing platforms, variant calling, etc. can introduce bias (i.e., batch effects) that result in confounding. Correcting for these confounders is critical and results based on the use of such population controls that lack explicit description of how these were managed should be interpreted with caution. Extensive considerations on the use sequence data from population controls are provided by Dercach, *et al* [40], along with methods for potential bias correction. Additionally, the large imbalance in samples sizes of the groups compared in the extreme phenotype vs. control population design must be considered. With very different sample sizes, tests that are based on the mean and variance of the test statistic for large samples (“second order asymptotics”, which is by far the most common measure of operating characteristics of a test) can fail to provide reliable p-values with test sizes that are too large (p-values that are too small). The aSKAT-O test with its “small sample adjustment” provided a correction for this problem. Permutation tests, including Fisher’s exact test, also retain reliability with imbalanced sample sizes but suffer from lower power, challenges with covariate adjustment and difficulty in computational implementation over the entire exome compared to aSKAT-O. Nevertheless, as data from thousands of exomes / genomes that can serve as controls become available to the scientific community, we think the extreme phenotype vs. population controls will become an increasingly popular study design.

## Materials and Methods

### Ethics statement

This research was approved by the Seattle Children's Hospital IRB (approval numbers 12974 and 11686). All study participants provided written informed consent or assent for the use of their DNA in studies aimed at identifying genetic risk variants for cystic fibrosis (CF) and for broad data sharing. Institutional certification was obtained for each sample in which exome sequencing was performed to allow deposition of phenotype and genotype data in the database for Genotypes and Phenotypes (dbGaP) and BAM files in the NCBI short-read archive.

### Exome sequencing

#### QC of sample DNA, library production, exome capture, clustering and sequencing

Detailed methods are given in Emond et al [16]. Initial quality control (QC) performed on all samples included sample quantification (PicoGreen), sex determination and genotype fingerprinting (using Illumina BeadXpress). Approximately 3.5 ug of genomic DNA was used library construction steps; sample shotgun libraries were captured for exome enrichment using one of three in-solution capture products: CCDS 2008 (~26 Mb), Roche/Nimblegen SeqCap EZ Human Exome Library v1.0 (~32 Mb; Roche Nimblegen EZ Cap v1) or EZ Cap v2 (~34 Mb). Using the automated Illumina cBot cluster station, non-multiplexed samples were processed in batches of eight (one for each lane of the flow-cell). Hybridization was followed by cluster generation via bridge PCR, and enriched libraries were sequenced on either an Illumina GAIIx or HiSeq2000.

### Read mapping, variant calling, and filtering

Detailed methods are provided in Emond et al [16]. Single nucleotide variants (SNVs) were called using the UMAKE pipeline at University of Michigan, which allowed all samples to be analyzed simultaneously, both for variant calling and filtering. BAM files were summarized using BWA, refined by duplicate removal, recalibration, and indel re-alignment. We excluded all reads that were not confidently mapped (Phred-scaled mapping quality < 20) from further analysis. To avoid PCR artifacts, we clipped overlapping ends in paired reads. We then computed genotype likelihoods for exome targeted regions and 50 flanking bases, accounting for per base alignment quality (BAQ) using samtools [41]. Variable sites and their allele frequencies were identified using glfMultiples, and a support vector machine (SVM) classifier was used flag probable false-positive variant sites. SNVs at HapMap polymorphic sites and Omni 2.5 array polymorphic sites in the 1000 Genomes project data were flagged as likely true positives. A total of 1,908,614 SNVs passed the SVM filter, with an overall transversion to transition ratio (Ts/Tv) of 2.84. After the initial SNV calls were generated, we re-examined the VCF files and applied filters considering total read depth, the number of individuals with coverage at the site, the fraction of variant reads in each heterozygote, the ratio of forward and reverse strand reads for reads carrying reference and variant alleles, and the average position of variant alleles along a read. CFTR genotypes from the exome sequencing results were in agreement with the clinical genotype data.

### Selection of phenotypic extremes

The definition of chronic *P*. *aeruginosa* infection was chosen to parallel the “Leeds” criterion suggested by Lee et al. within the sampling frame of the EPIC study. Routine bacterial cultures were scheduled to be taken during quarterly visits (once every 3-months). We defined an individual to have reached the chronic endpoint if s/he had at least two positive *P*. *aeruginosa* culture-quarters during any one-year period. Individuals in the analysis set had a median of 3.5 culture-quarters per year.

### Selection of extreme individuals

Individuals with extreme phenotypes were selected from the EPIC study, which was open to CF-affected individuals of any CFTR genotype, and from GMS, which included only individuals with the *F508del-CFTR* homozygous genotype. The early age-of-onset group comprised all individuals of European ancestry in either EPIC or GMS who had consented/assented for deposition of exome data to dbGaP and who had reached the chronic infection endpoint by age 7 at the time of exome sequencing (ancestry was determined using principal components analysis). Age 7 years demarks the earliest quartile of onset ages among EPIC DNA Collection study individuals (median age-of-onset of chronic *P*. *aeruginosa* airway infection = 14.3 years among the 1322 participants from EPIC DNA Collection study). Four individuals with age-of-onset later than 7 years but less than the median were included in the early onset extreme in order to make use of available sequencing, resulting in a total of 11 early onset individuals from GMS and 75 from EPIC. The median age-of-onset among the early extreme was 2.6 years.

EPIC individuals for the late onset extreme (n = 46 successful exomes) were selected from among the oldest individuals who were still free of chronic *P*. *aeruginosa* airway infection at the time of selection of the exome sequencing sample. The 19 individuals selected for the late onset extreme from the GMS were free of chronic *P*. *aeruginosa* airway infection until at least age 18 years, with one person free until age 58 years. One person in the late onset extreme became chronic at age 14.4 years after being selected for sequencing; otherwise, no person in the late onset extreme became chronically infected before age 21. The number of quarters with positive *P*. *aeruginosa* culture results among the early onset extreme was 70 times greater than that for the late onset extreme (905 vs 13) despite the longer observation times for the latter. The percentage of individuals in the early and late extremes who were identified by newborn screening (NBS) was 38.9% and 6.5%, respectively, compared to 21.6% for the EPIC Observational cohort overall [42]). Because NBS has a protective effect on acquisition of Pa [42], a larger percent of NBS individuals in the early-onset extreme makes this sample even more extreme in terms of Pa risk than it would be with a smaller percentage of NBS-identified individuals. Pancreatic enzyme use was similar in both extremes (93.4% early onset individuals ever used pancreatic enzymes, compared to 94.6% of the late onset individuals.)

### Two sample test by gene for exome data

The adjusted SKAT-O method described Lee et al. [20] was used to obtain p-values for each gene. Non-synonymous variants annotated to a given gene according to the RefSeq/NCBI 37/Hg19 model were included in each by-gene test. The small-sample adjustment was critical for the discovery analysis, as the imbalance in sample sizes resulted in marked overdispersion of the qq-plots (spuriously low p-values; S2 Fig) for tests without a small sample correction. Each by-gene test was adjusted for PC1, PC2 and PC3 from a principal components decomposition of the entire set of exome data after applying the QC filters described above; any control group individuals that could be potential ancestral outliers were liberally trimmed using smartPCA from the EIGENSOFT package (http://www.hsph.harvard.edu/alkes-price/software/). Powers for the extreme phenotypes design (n = 65 vs. n = 85) and the extreme phenotype vs. control large set design were calculated prior to the analysis assuming an elevated frequency of risk variants in one extreme and not the other; powers specific to *TMC6* were calculated post hoc by modeling the time-to-event distributions with Weibull distributions and by using the observed MAFs of variants in *TMC6*.

### Cox proportional hazards validation analyses

Censored data methods (Kaplan-Meier survival curves and the Cox proportional hazards (PH) model) were used to in the validation analysis to estimate age-of-onset of chronic *P*. *aeruginosa* airway infection and to test for differences between genetic groups in the associated hazard ratio (HR) for onset of chronic *P*. *aeruginosa* infection. The Cox PH model test has maximal statistical power when hazards are proportional, but the test size remains valid when the PH assumption is violated, providing an a priori parsimonious test choice in this time-to-event scenario. All models were stratified into five groups according to quintiles of enrollment age, because enrollment criteria for the validation set included being free of chronic *P*. *aeruginosa* airway infection, making it invalid to compare children enrolled at later ages (selected to be free of chronic *P*. *aeruginosa* airway infection) to those enrolled at earlier ages. Heuristically, stratification in the Cox model results in making comparisons within strata without the need for proportional hazards across strata, and then combines the results from different strata for an overall test statistic [43]. While over-stratification can result in a loss of power, failure to stratify could possibly lead to confounding, which is the more important consideration here. All models included enrollment age, the number of observations on study (in order to adjust for less sensitivity for detection of the end-point in individuals with fewer observations) and an indicator for *CFTR* risk group 2 (to adjust for severity of the CFTR dysfunction)[21]. The two group classification system of McKone *et al* is a parsimonious method for a complex set of genotypes that works well in practice: risk group 2 genotypes include those with at least one functional group IV or V allele, are thought to have residual CFTR function, correlate highly with pancreatic sufficient disease and have a generally milder course of disease [21], and was significantly associated with age-of-onset of chronic *P*. *aeruginosa* in all models (p ≤ 0.01). Sex/gender, pancreatic enzyme use, identification by newborn screening and *F508del-CFTR* homozygous genotype were not significant predictors of age-of-onset, did not affect variant HR estimates and were not included in the final models. Race was not included in the model because there were not enough individuals on non-European ancestry to attain convergence of the estimation algorithm. However, the models were fitted for individuals of European ancestry only to determine whether any significant effects were driven by the few African American individuals in the analysis (S1 Table). Further, principal components were constructed for the subset of validation individuals for whom chip data were available (N = 608), and PCs 1, 2, and 3 were used to adjust for possible confounding by ancestry among this subset of CF individuals. To even further avoid potential ancestral confounding, models were fitted to the subset of *F508del-CFTR* homozygous individuals, both with and without PC adjustment, to ensure that residual confounding by happenstance imbalance in CFTR-mutation severity among CAV2 or TMC6 groups did not account for the observed associations.

### Lung function analysis

As an additional validation analysis, association between discovered variants and lung function was tested. All EPIC participants underwent lung function tests starting at age 6 years of age as part of routine care, generally on a quarterly basis. Forced expiratory volume in 1 second (FEV1) is regarded as the most informative measure of lung function among CF individuals, and CF-specific percentile equations have been developed for lung function studies in CF. We tested for association between mean CF-specific FEV1 and *TMC6* and *CAV2* variants using generalized estimating equations (GEE) to account for correlation of measurements within subjects after adjusting for age and *CFTR* clinical risk group. Five hundred seventy-two children had variant calls and lung function measurements at age 7 and beyond (lung function measurements before age 7 not used due to the “learning” effect and potential unreliable measurements before age 7) with a median of 23 lung function measurements and a median of 4.4 years of observation time (min = .5, max = 12).

### Calculation of principal components

Principal components (PCs) were calculated for the 557 individuals with exome chip data using ancestry informative markers on the chip. These PCs were used to adjust for potential confounding by population stratification in the validation analysis. In addition, we sought to increase the sample size for the validation analysis with PC adjustment and to compare exome-based PC adjusted with AIMs-based PC adjustment by using genotype information from two different sources. Among the validation individuals who were genotyped for *CAV2* manually and who did not have chip data (n = 86), most (n = 51) had 353 ancestry informative markers (AIMs) available from the Illumina Golden Gate AIM (“fingerprint”) chip used in another study, and n = 354 individuals had data from both chips. Among subjects who had PCs for both the exome chip and the fingerprint chip, the linear correlation between PCs1 from either source was very high (r = 0.91) with higher correlation among individuals not clustering with the main group (r = 0.98). This indicates that one set of PCs can be converted to the other via a linear transformation for the purposes of identifying ancestral group. We regressed the exome chip PCs on the fingerprint chip PCs to obtain a linear conversion equation by which to convert PCs from the fingerprint chip to the scale of those for the exome chip. We converted PC1 for the 53 individuals without exome chip data to the exome chip scale for use in adjusting for ancestry in the validation analyses for sample size of 608. We examined all three **subsets** with PC data available: PCs from exome chip only, PCs from fingerprint chip only; and PCs from combined sources.

## Supporting Information

S1 Fig Differences in age-of-onset of chronic *Pa* infection between the two extreme phenotype samples used in this study are illustrated via Kaplan-Meier (KM) curves. The KM curve for age-of-onset among the 643 validation individuals is shown for comparison.
(DOCX)

S2 Fig Example QQ plot showing the results using SKAT without a small-sample adjustment for analysis of CF exomes (n = 86) versus >3000 ESP controls, all of European ancestry with PC adjustment included in the model. Lack of small-sample adjustment with imbalanced sample sizes leads to gross over-dispersion of the observed p-values, illustrating the importance of using a small-sample adjustment or a permutation-based test when the sample sizes are different.
(DOCX)

S3 Fig Age of onset of chronic *Pa* infection among delF508-*CFTR* homozygotes with the rs8940 ancestral (non-protective) allele. The estimated hazard ratio for children with the rs4312518 derived allele is 29.1 (p = 0.00072, 95% CI = [4.1, 205].) DelF508-*CFTR* homozygote children with the TMC6 rs4312518 derived allele and with at least one protective CAV2 allele (rs8940 derived allele) had HR = 2.6 (95% CI = [0.3, 23.1])(p = 0.03 for TMC6-CAV2 interaction within the F508del homozygous sub-group).
(DOCX)

S1 Table Demographic and clinical characteristics of the 643 individuals in the validation analysis for CAV2 with comparison by rs8940 allele group. FEVpp = CF-specific FEV1 percentile.
(DOCX)

S2 Table Cox model results for association of rs8940 allele groups with age-of-onset of chronic *P*. *aeruginosa* infection among 643 CF-affected validation individuals and relevant subsets. CAV2 rs8940 genotypes were obtained from the exome chip for 557 individuals who also had DCTN4 sequencing done by the Sanger method. Eighty-six additional individuals were manually genotyped for rs8940 and did not have chip data available for PC-adjustment. Ancestry informative markers (AIMs) were available for 406 individuals from another study, and we used the AIMs to determine whether the exome-chip-based PCs provided similar results compared to using AIMs. PC-adjustment method 1 combines calls from two chips to create principal components; PC method 2 employs PCs from the exome chip; PC method 3 employs PCs from the Ilumina AIMs chip. (HR = hazard ratio; p = p-value; LB and UB are the lower and upper bounds of the 95% confidence interval for the HR, respectively.)
(DOCX)

S3 Table CAV2 rs8940 genotypes and minor allele frequencies among different CFTR mutation groups. A highly significant difference in minor allele frequency is found for children with both mutations in functional classes I or II (MAF = 0.31) compared to those with mutations that are not both in functional classes I or II (MAF = 0.18; p = 0.00027). It is worth noting that this strong association between highly deleterious CFTR mutations is not readily explained by physical linkage, as the distance between the sites is ~1Mb and the estimated age of the F508del-CFTR mutation is 57,000 years. The estimated r^2^ (D’) between rs8940 and F508del-CFTR is 0.0016 (0.0014) in the ESP population.
(DOCX)

S4 Table TMC6 variants observed in the late-onset chronic *Pa* extreme, in ESP controls, and in the early-onset chronic *Pa* extreme for comparison. Variants represented on the Illumina exome chip were tested in the validation analysis: the three common variants with MAFs > 6% were tested individually, while the remaining variants, all rare, were combined in a burden test. The highlighted variant was significant in the validation analysis (p < 0.05) after adjusting for the multiple testing (four tests). The significance of rs34712518 was greater after including a time-by-genotype interaction (Fig 2B.)
(DOCX)

S5 Table Cox model results from the validation analysis of TMC6 rs34712518. The association of TMC6 rs34712518 with age-of-onset of chronic *P*a infection was found to increase with age, and an age-by-genotype interaction is included in all models in this table. (The primary validation test did not include the age interaction and results in a more conservative p-value (p = 0.012) and a hazard ratio that is averaged over all ages (HR = 1.8, [1.3–2.8])). The results from PC-adjustments and restriction to individuals who are homozygous for CFTRdelF508 show strong associations and large hazard ratios, indicating that the result is not due to confounding by ancestry nor by severity of CFTR mutation. A significant interaction was found between TMC6 rs34712518 and CAV2 rs8940, which is shown is this table by the low HR among children with a protective CAV2 allele (HR = 2.1, NS) and the high HR among children no CAV2 protective allele (HR = 12.1, p = 2.6x10^-5^). The HR was even larger in the analysis of children without the CAV2 protective allele when restricted to CFTR-F508del homozygotes (HR = 29.1; S3 Fig), but the difference between the last two models was not statistically significant. PC-adjustment method 2 employs PCs from the exome chip; PC method 3 employs PCs from the IIlumina AIMs chip. HR = hazard ratio; p = p-value; LB and UB are the lower and upper bounds of the 95% confidence interval for the HR, respectively. HRs are calculated at age 10.
(DOCX)

S1 Text Further acknowledgments. (DOCX)
