# Correction: Exome Sequencing of Phenotypic Extremes Identifies *CAV2* and *TMC6* as Interacting Modifiers of Chronic *Pseudomonas aeruginosa* Infection in Cystic Fibrosis

**DOI:** 10.1371/journal.pgen.1005424

**Published:** 2015-08-18

**Authors:** Mary J. Emond, Tin Louie, Julia Emerson, Jessica X. Chong, Rasika A. Mathias, Michael R. Knowles, Mark J. Rieder, Holly K. Tabor, Debbie A. Nickerson, Kathleen C. Barnes, NHLBI GO Exome Sequencing Project, Lung GO, Ronald L. Gibson, Michael J. Bamshad

In the Funding section, two of the grant numbers from the funder Cystic Fibrosis Foundation are listed incorrectly: OBSERV04KO should be OBSERV13K0, and BAMSHA14P should be BAMSHA14P0.
