# Supplementary material for: Exome Sequencing of Phenotypic Extremes Identifies CAV2 and TMC6 as Interacting Modifiers of Chronic Pseudomonas aeruginosa Infection in Cystic Fibrosis
Source: PLoS Genet. 2015 Jun 5;11(6):e1005273. doi: 10.1371/journal.pgen.1005273 (PMC4457883; doi:10.1371/journal.pgen.1005273)
Supplement: S1 Fig — The KM curve for age-of-onset among the 643 validation individuals is shown for comparison. (DOCX) [file pgen.1005273.s001.docx]

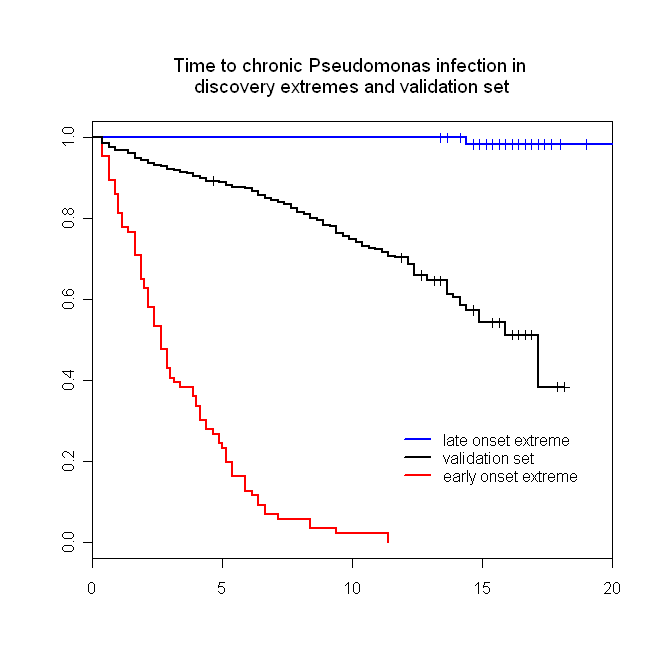


**Figure S1** – Differences in age-of-onset of chronic *Pa* infection between the two extreme phenotype samples used in this study are illustrated via Kaplan-Meier (KM) curves. The KM curve for age-of-onset among the 643 validation individuals is shown for comparison.
