# Supplementary material for: Exome Sequencing of Phenotypic Extremes Identifies CAV2 and TMC6 as Interacting Modifiers of Chronic Pseudomonas aeruginosa Infection in Cystic Fibrosis
Source: PLoS Genet. 2015 Jun 5;11(6):e1005273. doi: 10.1371/journal.pgen.1005273 (PMC4457883; doi:10.1371/journal.pgen.1005273)
Supplement: S2 Fig — Lack of small-sample adjustment with imbalanced sample sizes leads to gross over-dispersion of the observed p-values, illustrating the importance of using a small-sample adjustment or a permutation-based test when the sample sizes are different. (DOCX) [file pgen.1005273.s002.docx]

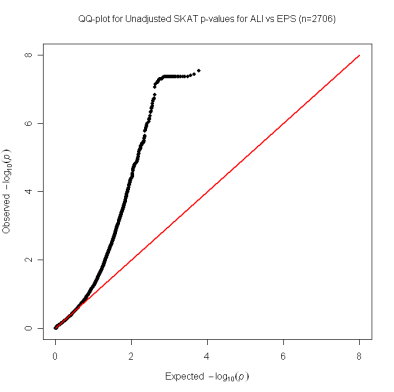


**Figure S2** – Example QQ plot showing the results using SKAT without a small-sample adjustment for analysis of CF exomes (n=86) versus >3000 ESP controls, all of European ancestry with PC adjustment included in the model. Lack of small-sample adjustment with imbalanced sample sizes leads to gross over-dispersion of the observed p-values, illustrating the importance of using a small-sample adjustment or a permutation-based test when the sample sizes are different.
