# Supplementary material for: Exome Sequencing of Phenotypic Extremes Identifies CAV2 and TMC6 as Interacting Modifiers of Chronic Pseudomonas aeruginosa Infection in Cystic Fibrosis
Source: PLoS Genet. 2015 Jun 5;11(6):e1005273. doi: 10.1371/journal.pgen.1005273 (PMC4457883; doi:10.1371/journal.pgen.1005273)
Supplement: S3 Fig — The estimated hazard ratio for children with the rs4312518 derived allele is 29.1 (p = 0.00072, 95% CI = [4.1, 205].) DelF508-CFTR homozygote children with the TMC6 rs4312518 derived allele and with at least one protective CAV2 allele (rs8940 derived allele) had HR = 2.6 (95% CI = [0.3, 23.1])(p = 0.03 for TMC6-CAV2 interaction within the F508del homozygous sub-group). (DOCX) [file pgen.1005273.s003.docx]

**
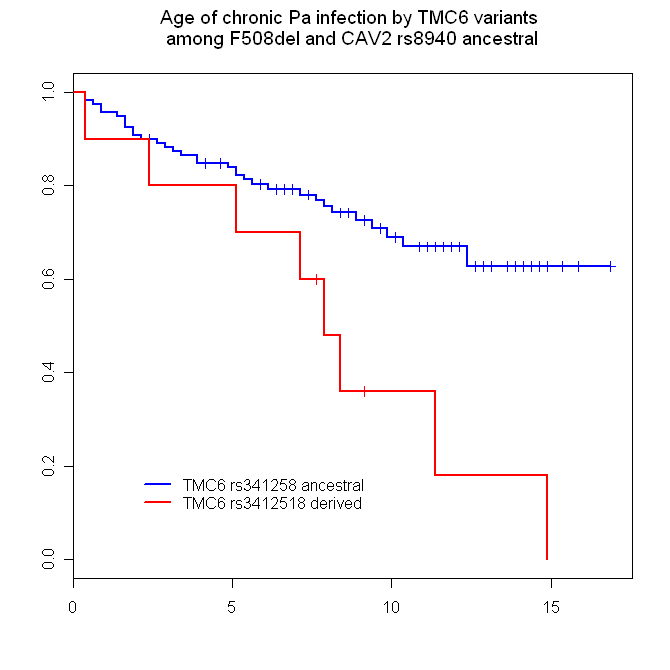
**

**Figure S3** - Age of onset of chronic *Pa* infection among delF508-*CFTR* homozygotes with the rs8940 ancestral (non-protective) allele. The estimated hazard ratio for children with the rs4312518 derived allele is 29.1 (p=0.00072, 95% CI=[4.1, 205].) DelF508-*CFTR* homozygote children with the TMC6 rs4312518 derived allele and with at least one protective CAV2 allele (rs8940 derived allele) had HR =2.6 (95% CI=[0.3, 23.1])(p=0.03 for TMC6-CAV2 interaction within the F508del homozygous sub-group).
