# Supplementary material for: Exome Sequencing of Phenotypic Extremes Identifies CAV2 and TMC6 as Interacting Modifiers of Chronic Pseudomonas aeruginosa Infection in Cystic Fibrosis
Source: PLoS Genet. 2015 Jun 5;11(6):e1005273. doi: 10.1371/journal.pgen.1005273 (PMC4457883; doi:10.1371/journal.pgen.1005273)
Supplement: S1 Table — FEVpp = CF-specific FEV1 percentile. (DOCX) [file pgen.1005273.s004.docx]

|  | **all subjects** | **rs8940 ancestral** | **rs8940 alternate** |
| --- | --- | --- | --- |
| Number in group | 643 | 326 | 317 |
| Age (yrs) at diagnosis of CF, mean (SD) | 0.97 (1.83) | 0.97 (1.87) | 0.98 (1.80) |
| Number of qtrs. with Pa culture, median (range) | 23 (6, 51) | 23 (6, 50) | 23 (7, 51) |
| Mean FEVpp over year leading up to endpoint^1^, mean (SD) | n=429 0.661 (0.211) | n=207 0.646 (0.205) | n=222 0.676 (0.216) |
| Number (%) male | 311 (48.4) | 167 (53.7) | 144 (46.3) |
| White non-Hispanic,   number (%) | 595 (92.5) | 300 (92.0) | 295 (93.1) |
| White Hispanic,  number (%) | 21 (3.3) | 14 (4.3) | 7 (2.2) |
| African American (%) | 20 (3.1) | 9 (2.8) | 11 (3.5) |
| Asian (%) | 3 (0.5) | 2 (0.6) | 1 (0.3) |
| Aleut (%) | 3 (0.5) | 1 (0.3) | 2 (0.6) |
| Other (%) | 1 (0.2) | 0 (0.0) | 1 (0.3) |
| Age (yrs) at data freeze, median (range) | 8.6 (2.4, 17.9) | 8.6 (2.4, 17.4) | 8.6 (3.9, 17.9) |
| dF508 homozygous,  number (%) | 312 (48.5) | 151 (46.3) | 161 (50.8) |
| CFTR clinical risk group 1,  number (%) | 492 (76.5) | 239 (73.3) | 253 (79.8) |
| Sweat chloride mEq/L,  mean (SD) | 97.32 (20.27) | 97.14 (20.65) | 97.50 (19.92) |
| Meconium ileus at birth,  number (%) | 138 (21.5) | 71 (21.8) | 67 (21.1) |
| Diagnosed by newborn  screening | N=625  137 (21.9) | n=308  70 (22.7) | n=317  67 (21.1) |

**^1^Or mean FEVpp one year leading up to last Pa culture for individuals not reaching the endpoint**

**Table S1** – Demographic and clinical characteristics of the 643 individuals in the validation analysis for CAV2 with comparison by rs8940 allele group. FEVpp=CF-specific FEV1 percentile.
