# Supplementary material for: Exome Sequencing of Phenotypic Extremes Identifies CAV2 and TMC6 as Interacting Modifiers of Chronic Pseudomonas aeruginosa Infection in Cystic Fibrosis
Source: PLoS Genet. 2015 Jun 5;11(6):e1005273. doi: 10.1371/journal.pgen.1005273 (PMC4457883; doi:10.1371/journal.pgen.1005273)
Supplement: S2 Table — CAV2 rs8940 genotypes were obtained from the exome chip for 557 individuals who also had DCTN4 sequencing done by the Sanger method. Eighty-six additional individuals were manually genotyped for rs8940 and did not have chip data available for PC-adjustment. Ancestry informative markers (AIMs) were available for 406 individuals from another study, and we used the AIMs to determine whether the exome-chip-based PCs provided similar results compared to using AIMs. PC-adjustment method 1 combines calls from two chips to create principal components; PC method 2 employs PCs from the exome chip; PC method 3 employs PCs from the Ilumina AIMs chip. (HR = hazard ratio; p = p-value; LB and UB are the lower and upper bounds of the 95% confidence interval for the HR, respectively.) (DOCX) [file pgen.1005273.s005.docx]

| **Model** | **N** | **rs8940 Group** | **n** | **HR** | **p** | **LB** | **UB** |
| --- | --- | --- | --- | --- | --- | --- | --- |
|  |  |  |  |  |  |  |  |
| Primary | 643 | ancestral | 326 |  |  |  |  |
|  |  | derived | 317 | 0.53 | 0.012 | 0.32 | 0.88 |
| PC-adjusted, method 1 | 608 | ancestral | 306 |  |  |  |  |
|  |  | derived | 302 | 0.48 | 0.0048 | 0.29 | 0.8 |
| PC-adjusted, method 2 | 557 | ancestral | 276 |  |  |  |  |
|  |  | derived | 280 | 0.49 | 0.0099 | 0.29 | 0.84 |
| PC-adjusted, method 3 | 406 | ancestral | 216 |  |  |  |  |
|  |  | derived | 190 | 0.36 | 0.0044 | 0.18 | 0.73 |
| F508∆CFTR only | 321 | ancestral | 151 |  |  |  |  |
|  |  | derived | 171 | 0.44 | 0.027 | 0.21 | 0.91 |
| F508∆CFTR white/PC-adjusted | 268 | ancestral | 118 |  |  |  |  |
|  |  | derived | 150 | 0.35 | 0.0087 | 0.16 | 0.77 |

**Table S2** – Cox model results for association of rs8940 allele groups with age-of-onset of chronic *P. aeruginosa* infection among 643 CF-affected validation individuals and relevant subsets. CAV2 rs8940 genotypes were obtained from the exome chip for 557 individuals who also had DCTN4 sequencing done by the Sanger method. Eighty-six additional individuals were manually genotyped for rs8940 and did not have chip data available for PC-adjustment. Ancestry informative markers (AIMs) were available for 406 individuals from another study, and we used the AIMs to determine whether the exome-chip-based PCs provided similar results compared to using AIMs. PC-adjustment method 1 combines calls from two chips to create principal components; PC method 2 employs PCs from the exome chip; PC method 3 employs PCs from the Ilumina AIMs chip. (HR=hazard ratio; p=p-value; LB and UB are the lower and upper bounds of the 95% confidence interval for the HR, respectively.)
