# Supplementary material for: Exome Sequencing of Phenotypic Extremes Identifies CAV2 and TMC6 as Interacting Modifiers of Chronic Pseudomonas aeruginosa Infection in Cystic Fibrosis
Source: PLoS Genet. 2015 Jun 5;11(6):e1005273. doi: 10.1371/journal.pgen.1005273 (PMC4457883; doi:10.1371/journal.pgen.1005273)
Supplement: S3 Table — A highly significant difference in minor allele frequency is found for children with both mutations in functional classes I or II (MAF = 0.31) compared to those with mutations that are not both in functional classes I or II (MAF = 0.18; p = 0.00027). It is worth noting that this strong association between highly deleterious CFTR mutations is not readily explained by physical linkage, as the distance between the sites is ~1Mb and the estimated age of the F508del-CFTR mutation is 57,000 years. The estimated r2 (D’) between rs8940 and F508del-CFTR is 0.0016 (0.0014) in the ESP population. (DOCX) [file pgen.1005273.s006.docx]

|  | **N** | **rs8940 ancestral hmz** | **rs8940 het** | **rs8940 derived hmz** | **MAF** |
| --- | --- | --- | --- | --- | --- |
| both mutations are class I: | 10 | 7 | 2 | 1 | 0.2 |
| both are class I or class II but not dF508 | 14 | 8 | 5 | 1 | 0.25 |
| both are class I or class II | 452 | 210 | 200 | 40 | 0.31 |
| not both are class I or class II | 109 | 74 | 30 | 5 | 0.183 |
| dF508 Homozygotes | 323 | 157 | 132 | 34 | 0.31 |
| N1303K Hets | 21 | 7 | 13 | 1 | 0.357 |
| N1303K + dF508 | 14 | 5 | 9 | 0 | 0.32 |
| N1303K, no dF508 | 7 | 2 | 4 | 1 | 0.429 |
| N1303K homozygotes (none) |  |  |  |  |  |
| G542X hets | 25 | 10 | 13 | 2 | 0.34 |
| G542X + dF508 | 14 | 6 | 7 | 1 | 0.32 |
| G542X, no dF508 | 11 | 4 | 6 | 1 | 0.363 |
| W1282X hets | 9 | 3 | 6 | 0 | 0.333 |
| W1282X + dF508 | 4 | 2 | 2 | 0 | 0.25 |
| W1282X, no dF508 | 5 | 1 | 4 | 0 | 0.4 |
| N1303K or W1282X, no dF508 | 11 | 3 | 7 | 1 | 0.41 |

**Table S3 -** CAV2 rs8940 genotypes and minor allele frequencies among different CFTR mutation groups. A highly significant difference in minor allele frequency is found for children with both mutations in functional classes I or II (MAF=0.31) compared to those with mutations that are not both in functional classes I or II (MAF = 0.18; p=0.00027). It is worth noting that this strong association between highly deleterious CFTR mutations is not readily explained by physical linkage, as the distance between the sites is ~1Mb and the estimated age of the F508del-CFTR mutation is 57,000 years. The estimated r^2^ (D’) between rs8940 and F508del-CFTR is 0.0016 (0.0014) in the ESP population.
