# Supplementary material for: Exome Sequencing of Phenotypic Extremes Identifies CAV2 and TMC6 as Interacting Modifiers of Chronic Pseudomonas aeruginosa Infection in Cystic Fibrosis
Source: PLoS Genet. 2015 Jun 5;11(6):e1005273. doi: 10.1371/journal.pgen.1005273 (PMC4457883; doi:10.1371/journal.pgen.1005273)
Supplement: S4 Table — Variants represented on the Illumina exome chip were tested in the validation analysis: the three common variants with MAFs > 6% were tested individually, while the remaining variants, all rare, were combined in a burden test. The highlighted variant was significant in the validation analysis (p < 0.05) after adjusting for the multiple testing (four tests). The significance of rs34712518 was greater after including a time-by-genotype interaction (Fig 2B.) (DOCX) [file pgen.1005273.s007.docx]

| **position** | **rs** | **On exome chip** | **observed MAF in late onset extreme** | **ESP MAF** | **Observed MAF in early onset extreme** |
| --- | --- | --- | --- | --- | --- |
| 76109689 | rs141422203 |  | 0.00% | 0.02% | 0.00% |
| 76113383 | unknown |  | 0.00% | 0.02% | 0.00% |
| 76113652 | unknown |  | 0.00% | 0.03% | 0.00% |
| 76113911 | rs148112314 |  | 0.00% | 0.02% | 0.00% |
| 76115096 | rs149875354 |  | 0.00% | 0.02% | 0.00% |
| 76115102 | rs149053739 |  | 0.00% | 0.02% | 0.00% |
| 76115435 | unknown | 1 | 0.00% | 0.02% | 0.00% |
| 76115436 | unknown |  | 0.00% | 0.02% | 0.00% |
| 76115441 | rs147815166 | 1 | 0.00% | 0.08% | 1.16% |
| 76116750 | unknown |  | 0.00% | 0.02% | 0.00% |
| 76116857 | rs146998467 | 1 | 0.00% | 0.03% | 0.00% |
| 76116858 | rs139049167 |  | 0.00% | 0.02% | 0.00% |
| 76117124 | rs75400929 | 1 | 0.00% | 0.68% | 0.58% |
| 76117173 | unknown |  | 0.00% | 0.02% | 0.00% |
| 76117672 | rs138918101 |  | 0.00% | 0.02% | 0.00% |
| 76118694 | unknown |  | 0.00% | 0.02% | 0.00% |
| 76118700 | unknown |  | 0.00% | 0.02% | 0.00% |
| 76118774 | rs147856997 | 1 | 0.00% | 0.02% | 0.00% |
| 76118775 | unknown |  | 0.00% | 0.02% | 0.00% |
| 76118810 | rs139624494 |  | 0.00% | 0.02% | 0.00% |
| 76120102 | rs149107165 |  | 0.00% | 0.02% | 0.00% |
| 76120169 | unknown |  | 0.00% | 0.02% | 0.00% |
| 76120606 | unknown | 1 | 0.00% | 0.05% | 0.00% |
| 76120649 | unknown |  | 0.00% | 0.09% | 0.00% |
| 76120658 | rs146371375 | 1 | 0.00% | 0.02% | 0.00% |
| 76120673 | rs138860970 |  | 0.00% | 0.02% | 0.00% |
| 76120771 | unknown |  | 0.00% | 0.02% | 0.00% |
| 76120993 | rs139654750 | 1 | 0.00% | 0.08% | 0.00% |
| 76121004 | rs9895373 | 1 | 0.00% | 0.23% | 0.00% |
| 76121031 | rs34712518 | 1 | 1.79% | 6.27% | 7.14% |
| 76121318 | rs12449858 | 1 | 14.62% | 9.48% | 8.14% |
| 76121864 | rs2748427 | 1 | 30.77% | 19.34% | 20.35% |
| 76121920 | rs146371375 | 1 | 0.00% | 0.11% | 0.00% |
| 76121927 | rs117065924 | 1 | 0.00% | 0.04% | 0.00% |
| 76122692 | rs139636418 |  | 0.00% | 0.03% | 0.00% |
| 76122712 | unknown |  | 0.82% | 0.00% | 0.00% |

**Table S4** – TMC6 variants observed in the late-onset chronic *Pa* extreme, in ESP controls, and in the early-onset chronic *Pa* extreme for comparison. Variants represented on the Illumina exome chip were tested in the validation analysis: the three common variants with MAFs > 6% were tested individually, while the remaining variants, all rare, were combined in a burden test. The highlighted variant was significant in the validation analysis (p < 0.05) after adjusting for the multiple testing (four tests). The significance of rs34712518 was greater after including a time-by-genotype interaction (Figure 2B.)
