# Supplementary material for: Exome Sequencing of Phenotypic Extremes Identifies CAV2 and TMC6 as Interacting Modifiers of Chronic Pseudomonas aeruginosa Infection in Cystic Fibrosis
Source: PLoS Genet. 2015 Jun 5;11(6):e1005273. doi: 10.1371/journal.pgen.1005273 (PMC4457883; doi:10.1371/journal.pgen.1005273)
Supplement: S5 Table — The association of TMC6 rs34712518 with age-of-onset of chronic Pa infection was found to increase with age, and an age-by-genotype interaction is included in all models in this table. (The primary validation test did not include the age interaction and results in a more conservative p-value (p = 0.012) and a hazard ratio that is averaged over all ages (HR = 1.8, [1.3–2.8])). The results from PC-adjustments and restriction to individuals who are homozygous for CFTRdelF508 show strong associations and large hazard ratios, indicating that the result is not due to confounding by ancestry nor by severity of CFTR mutation. A significant interaction was found between TMC6 rs34712518 and CAV2 rs8940, which is shown is this table by the low HR among children with a protective CAV2 allele (HR = 2.1, NS) and the high HR among children no CAV2 protective allele (HR = 12.1, p = 2.6x10^-5^). The HR was even larger in the analysis of children without the CAV2 protective allele when restricted to CFTR-F508del homozygotes (HR = 29.1; S3 Fig), but the difference between the last two models was not statistically significant. PC-adjustment method 2 employs PCs from the exome chip; PC method 3 employs PCs from the IIlumina AIMs chip. HR = hazard ratio; p = p-value; LB and UB are the lower and upper bounds of the 95% confidence interval for the HR, respectively. HRs are calculated at age 10. (DOCX) [file pgen.1005273.s008.docx]

| **Model** | **N** | **rs34712518 group** | **n** | **HR** | **p** | **LB** | **UB** |
| --- | --- | --- | --- | --- | --- | --- | --- |
| Primary | 579 | ancestral | 523 |  |  |  |  |
|  |  | derived | 56 | 5.4 | 0.0003 | 2.2 | 13.5 |
| PC-adjusted, method 2 | 579 | ancestral | 523 |  |  |  |  |
|  |  | derived | 56 | 5.5 | 0.0003 | 2.2 | 13.6 |
| PC-adjusted, method 3 | 359 | ancestral | 324 |  |  |  |  |
|  |  | derived | 35 | 4.3 | 0.01 | 1.4 | 13.4 |
| F508∆CFTR only | 273 | ancestral | 166 |  |  |  |  |
|  |  | derived | 19 | 7.7 | 0.0012 | 2.2 | 26.3 |
| F508∆CFTR white/PC-adjusted | 174 | ancestral | 158 |  |  |  |  |
|  |  | derived | 16 | 4.9 | 0.05 | 1.1 | 23.8 |
| among persons with at least one rs8940 derived (protective) allele | 288 | ancestral | 258 |  |  |  |  |
|  |  | derived | 30 | 2.1 | 0.41 | 0.34 | 11.3 |
| among persons with two rs8940 ancestral alleles | 291 | ancestral | 265 |  |  |  |  |
|  |  | derived | 26 | 12.1 | 2.6x10^-5^ | 3.8 | 38.8 |
| among F508∆CFTR homozygotes with two rs8940 ancestral alleles | 129 | ancestral | 119 |  |  |  |  |
|  |  | derived | 10 | 29.1 | 0.00072 | 4.1 | 205 |

**Table S5** – Cox model results from the validation analysis of TMC6 rs34712518. The association of TMC6 rs34712518 with age-of-onset of chronic *P*a infection was found to increase with age, and an age-by-genotype interaction is included in all models in this table. (The primary validation test did not include the age interaction and results in a more conservative p-value (p=0.012) and a hazard ratio that is averaged over all ages (HR=1.8, [1.3-2.8])). The results from PC-adjustments and restriction to individuals who are homozygous for CFTRdelF508 show strong associations and large hazard ratios, indicating that the result is not due to confounding by ancestry nor by severity of CFTR mutation. A significant interaction was found between TMC6 rs34712518 and CAV2 rs8940, which is shown is this table by the low HR among children with a protective CAV2 allele (HR=2.1, NS) and the high HR among children no CAV2 protective allele (HR = 12.1, p=2.6x10^-5^). The HR was even larger in the analysis of children without the CAV2 protective allele when restricted to CFTR-F508del homozygotes (HR=29.1; Figure S3), but the difference between the last two models was not statistically significant. PC-adjustment method 2 employs PCs from the exome chip; PC method 3 employs PCs from the IIlumina AIMs chip. HR=hazard ratio; p=p-value; LB and UB are the lower and upper bounds of the 95% confidence interval for the HR, respectively. HRs are calculated at age 10.
