# Supplementary material for: Exome Sequencing of Phenotypic Extremes Identifies CAV2 and TMC6 as Interacting Modifiers of Chronic Pseudomonas aeruginosa Infection in Cystic Fibrosis
Source: PLoS Genet. 2015 Jun 5;11(6):e1005273. doi: 10.1371/journal.pgen.1005273 (PMC4457883; doi:10.1371/journal.pgen.1005273)
Supplement: S1 Text — (DOCX) [file pgen.1005273.s009.docx]

**Further acknowledgements**

**HeartGO:**

**Atherosclerosis Risk in Communities (ARIC)**: NHLBI (N01 HC-55015, N01 HC-55016, N01HC-55017, N01 HC-55018, N01 HC-55019, N01 HC-55020, N01 HC-55021); **Cardiovascular Health Study (CHS)**: NHLBI (HHSN268201200036C, N01-HC-85239, N01-HC-85079 through N01-HC-85086, N01-HC-35129, N01 HC-15103, N01 HC-55222, N01-HC-75150, N01-HC-45133, and grant HL080295), with additional support from NINDS and from NIA (AG-023629, AG-15928, AG-20098, and AG-027058); **Coronary Artery Risk Development in Young Adults (CARDIA)**: NHLBI (N01-HC95095 & N01-HC48047, N01-HC48048, N01-HC48049, and N01-HC48050); **Framingham Heart Study (FHS)**: NHLBI (N01-HC-25195 and grant R01 NS17950) with additional support from NIA (AG08122 and AG033193); **Jackson Heart Study (JHS)**: NHLBI and the National Institute on Minority Health and Health Disparities (N01 HC-95170, N01 HC-95171 and N01 HC-95172); **Multi-Ethnic Study of Atherosclerosis (MESA**)**:** NHLBI (N01-HC-95159 through N01-HC-95169 and RR-024156).

**Lung GO:**

**Cystic Fibrosis (CF):** Cystic Fibrosis Foundation (GIBSON07K0, KNOWLE00A0, OBSERV04K0, RDP R026), the NHLBI (R01 HL-068890, R02 HL-095396), NIH National Center for Research Resources (UL1 RR-025014), and the National Human Genome Research Institute (NHGRI) (5R00 HG-004316). **Chronic Obstructive Pulmonary Disease (COPDGene):** NHLBI (U01 HL-089897, U01 HL-089856), and the COPD Foundation through contributions made to an Industry Advisory Board comprised of AstraZeneca, Boehringer Ingelheim, Novartis, Pfizer, and Sunovian. The COPDGene clinical centers and investigators are available at www.copdgene.org. **Acute Lung Injury (ALI)**: NHLBI (RC2 HL-101779). **Lung Health Study (LHS)**: NHLBI (RC2 HL-066583), the NHGRI (HG-004738), and the NHLBI Division of Lung Diseases (HR-46002). **Pulmonary Arterial Hypertension (PAH):** NIH (P50 HL-084946, K23 AR-52742), and the NHLBI (F32 HL-083714). **Asthma:** NHLBI (RC2 HL-101651), and the NIH (HL-077916, HL-69197, HL-76285, M01 RR-07122).

**SWISS and ISGS:**

Siblings with Ischemic Stroke Study (SWISS): National Institute of Neurological Disorders and Stroke (NINDS) (R01 NS039987); Ischemic Stroke Genetics Study (ISGS): NINDS (R01 NS042733)

**WHISP:**

**Women’s Health Initiative (WHI):** The WHI Sequencing Project is funded by the NHLBI (HL-102924) as well as the National Institutes of Health (NIH), U.S. Department of Health and Human Services through contracts N01WH22110, 24152, 32100-2, 32105-6, 32108-9, 32111-13, 32115, 32118-32119, 32122, 42107-26, 42129-32, and 44221, and HHSN268201100046C. The authors thank the WHI investigators and staff for their dedication, and the study participants for making the program possible. A full listing of WHI investigators can be found at: <https://cleo.whi.org/researchers/Documents%20%20Write%20a%20Paper/WHI%20Investigator%20Short%20List.pdf>

**NHLBI GO Exome Sequencing Project**

**BroadGO**

Stacey B. Gabriel (Broad Institute)^4, 5, 11, 16, 17^, David M. Altshuler (Broad Institute, Harvard Medical School, Massachusetts General Hospital)^1, 5, 7, 17^, Gonçalo R. Abecasis (University of Michigan)^3, 5, 9, 13, 15, 17^, Hooman Allayee (University of Southern California)^5^, Sharon Cresci (Washington University School of Medicine)^5^, Mark J. Daly (Broad Institute, Massachusetts General Hospital), Paul I. W. de Bakker (Broad Institute, Harvard Medical School, University Medical Center Utrecht)^3, 15^, Mark A. DePristo (Broad Institute)^4, 13, 15, 16^, Ron Do (Broad Institute)^5, 9, 13, 15^, Peter Donnelly (University of Oxford)^5^, Deborah N. Farlow (Broad Institute)^3, 4, 5, 12, 14, 16, 17^, Tim Fennell (Broad Institute), Kiran Garimella (University of Oxford)^4, 16^, Stanley L. Hazen (Cleveland Clinic)^5^, Youna Hu (University of Michigan)^3, 9, 15^, Daniel M. Jordan (Harvard Medical School, Harvard University)^13^, Goo Jun (University of Michigan)^13^, Sekar Kathiresan (Broad Institute, Harvard Medical School, Massachusetts General Hospital)^5, 8, 9, 12, 14, 15, 17, 20^, Hyun Min Kang (University of Michigan)^9, 13, 16^, Adam Kiezun (Broad Institute)^5, 13, 15^, Guillaume Lettre (Broad Institute, Montreal Heart Institute, Université de Montréal)^1, 2, 13, 15^, Bingshan Li (University of Michigan)^3^, Mingyao Li (University of Pennsylvania)^5^, Christopher H. Newton-Cheh (Broad Institute, Massachusetts General Hospital, Harvard Medical School)^3, 8, 15^, Sandosh Padmanabhan (University of Glasgow School of Medicine)^3, 12, 15^, Gina Peloso (Broad Institute, Harvard Medical School, Massachusetts General Hospital)^5^, Sara Pulit (Broad Institute)^3, 15^, Daniel J. Rader (University of Pennsylvania)^5^, David Reich (Broad Institute, Harvard Medical School)^15^, Muredach P. Reilly (University of Pennsylvania)^5^, Manuel A. Rivas (Broad Institute, Massachusetts General Hospital)^5^, Steve Schwartz (Fred Hutchinson Cancer Research Center)^5, 12^, Laura Scott (University of Michigan)^1^, David S. Siscovick (University of Washington)^5, 1, 25^, John A. Spertus (University of Missouri Kansas City)^5^, Nathan O. Stitziel (Brigham and Women's Hospital)^5, 15^, Nina Stoletzki (Brigham and Women's Hospital, Broad Institute, Harvard Medical School)^13^, Shamil R. Sunyaev (Brigham and Women's Hospital, Broad Institute, Harvard Medical School)^1, 3, 5, 13, 15^, Benjamin F. Voight (Broad Institute, Massachusetts General Hospital), Cristen J. Willer (University of Michigan)^1, 9, 13, 15^

**HeartGO**

Stephen S. Rich (University of Virginia)^2, 4, 7, 8, 9, 11,14, 15, 17, 18, 31^, Ermeg Akylbekova (Jackson State University, University of Mississippi Medical Center)^29^, Larry D. Atwood* (Boston University)^1, 11, 28^, Christie M. Ballantyne (Baylor College of Medicine, Methodist DeBakey Heart Center)^9, 22^, Maja Barbalic (University of Texas Health Science Center Houston)^9, 14, 15, 17, 22^, R. Graham Barr (Columbia University Medical Center)^10, 31^, Emelia J. Benjamin (Boston University)^14, 20, 28^, Joshua Bis (University of Washington)^15, 23^, Eric Boerwinkle (University of Texas Health Science Center Houston)^3, 5, 9, 13, 15, 17, 22^, Donald W. Bowden (Wake Forest University)^1, 31^, Jennifer Brody (University of Washington)^3, 5, 15, 23^, Matthew Budoff (Harbor-UCLA Medical Center)^31^, Greg Burke (Wake Forest University)^5, 31^, Sarah Buxbaum (Jackson State University)^3, 13, 15, 29^, Jeff Carr (Wake Forest University)^25, 29, 31^, Donna T. Chen (University of Virginia)^6, 11^, Ida Y. Chen (Cedars-Sinai Medical Center)^1, 31^, Wei-Min Chen (University of Virginia)^13, 15, 18^, Pat Concannon (University of Virginia)^11^, Jacy Crosby (University of Texas Health Science Center Houston)^22^, L. Adrienne Cupples (Boston University)^1, 3, 5, 9, 13, 15, 18, 28^, Ralph D'Agostino (Boston University)^28^, Anita L. DeStefano (Boston University)^13, 18^, ^28^, Albert Dreisbach (University of Mississippi Medical Center)^3, 29^, Josée Dupuis (Boston University)^1, 28^, J. Peter Durda (University of Vermont)^15, 23^, Jaclyn Ellis (University of North Carolina Chapel Hill)^1^, Aaron R. Folsom (University of Minnesota)^5^, ^22^, Myriam Fornage (University of Texas Health Science Center Houston)^3, 18, 25^, Caroline S. Fox (National Heart, Lung, and Blood Institute)^1, 28^, Ervin Fox (University of Mississippi Medical Center)^3, 9, 29^, Vincent Funari (Cedars-Sinai Medical Center)^1, 11, 31^, Santhi K. Ganesh (University of Michigan)^2, 22^, Julius Gardin (Hackensack University Medical Center)^25^, David Goff (Wake Forest University)^25^, Ora Gordon (Cedars-Sinai Medical Center)^11, 31^, Wayne Grody (University of California Los Angeles)^11, 31^, Myron Gross (University of Minnesota)^1, 5, 14, 25^, Xiuqing Guo (Cedars-Sinai Medical Center)^3, 15, 31^, Ira M. Hall (University of Virginia), Nancy L. Heard-Costa (Boston University)^1, 11, 28^, Susan R. Heckbert (University of Washington)^10, 14, 20, 23^, Nicholas Heintz (University of Vermont), David M. Herrington (Wake Forest University)^5, 31^, DeMarc Hickson (Jackson State University, University of Mississippi Medical Center)^29^, Jie Huang (National Heart, Lung, and Blood Institute)^5, 28^, Shih-Jen Hwang (Boston University, National Heart, Lung, and Blood Institute)^3, 28^, David R. Jacobs (University of Minnesota)^25^, Nancy S. Jenny (University of Vermont)^1, 2, 23^, Andrew D. Johnson (National Heart, Lung, and Blood Institute)^2, 5, 11, 28^, Craig W. Johnson (University of Washington)^15, 31^, Steven Kawut (University of Pennsylvania)^10,31^, Richard Kronmal (University of Washington)^31^, Raluca Kurz (Cedars-Sinai Medical Center)^11, 31^, Ethan M. Lange (University of North Carolina Chapel Hill)^3, 5, 9, 13, 34^, Leslie A. Lange (University of North Carolina Chapel Hill)^1, 2, 3, 5, 9, 12, 13, 15, 17, 18, 20, 25, 34^, Martin G. Larson (Boston University)^3, 15, 28^, Mark Lawson (University of Virginia), Cora E. Lewis (University of Alabama at Birmingham)^25,34^, Daniel Levy (National Heart, Lung, and Blood Institute)^3, 15, 17, 28^, Dalin Li (Cedars-Sinai Medical Center)^11, 15, 31^, Honghuang Lin (Boston University)^20, 28^, Chunyu Liu (National Heart, Lung, and Blood Institute)^3, 28^, Jiankang Liu (University of Mississippi Medical Center)^1, 29^, Kiang Liu (Northwestern University)^25^, Xiaoming Liu (University of Texas Health Science Center Houston)^15, 22^, Yongmei Liu (Wake Forest University)^2, 5, 31^, William T. Longstreth (University of Washington)^18, 23^, Cay Loria (National Heart, Lung, and Blood Institute)^25^, Thomas Lumley (University of Auckland)^9, 23^, Kathryn Lunetta (Boston University)^28^, Aaron J. Mackey (University of Virginia)^16, 18^, Rachel Mackey (University of Pittsburgh)^1, 23, 31^, Ani Manichaikul (University of Virginia)^8, 15, 18, 31^, Taylor Maxwell (University of Texas Health Science Center Houston)^22^, Barbara McKnight (University of Washington)^15, 23^, James B. Meigs (Brigham and Women's Hospital, Harvard Medical School, Massachusetts General Hospital)^1, 28^, Alanna C. Morrison (University of Texas Health Science Center Houston)^3, 15, 17^, Solomon K. Musani (University of Mississippi Medical Center)^3, 29^, Josyf C. Mychaleckyj (University of Virginia)^13, 15, 31^, Jennifer A. Nettleton (University of Texas Health Science Center Houston)^9, 22^, Kari North (University of North Carolina Chapel Hill)^1, 3, 9, 10, 13, 15, 17, 34^, Christopher J. O'Donnell (Massachusetts General Hospital, National Heart, Lung, and Blood Institute)^2, 5, 9, 11, 12, 14, 15, 17, 20, 28^, Daniel O'Leary (Tufts University School of Medicine)^25, 31^, Frank S. Ong (Cedars-Sinai Medical Center)^3, 11, 31^, Walter Palmas (Columbia University)^3, 15, 31^, James S. Pankow (University of Minnesota)^1, 22^, Nathan D. Pankratz (Indiana University School of Medicine)^15, 25^, Shom Paul (University of Virginia), Marco Perez (Stanford University School of Medicine), Sharina D. Person (University of Alabama at Birmingham, University of Alabama at Tuscaloosa)^25^, Joseph Polak (Tufts University School of Medicine)^31^, Wendy S. Post (Johns Hopkins University)^3, 9, 11, 14, 20, 31^, Bruce M. Psaty (Group Health Research Institute, University of Washington)^3, 5, 9, 11, 14, 15, 23^, Aaron R. Quinlan (University of Virginia)^18, 19^, Leslie J. Raffel (Cedars-Sinai Medical Center)^6, 11, 31^, Vasan S. Ramachandran (Boston University)^3, 28^, Alexander P. Reiner (Fred Hutchinson Cancer Research Center, University of Washington)^1, 2, 3, 5, 9, 11, 12, 13, 14, 15, 20, 25, 34^, Kenneth Rice (University of Washington)^15, 23^, Jerome I. Rotter (Cedars-Sinai Medical Center)^1, 3, 6, 8, 11, 15, 31^, Jill P. Sanders (University of Vermont)^23^, Pamela Schreiner (University of Minnesota)^25^, Sudha Seshadri (Boston University)^18, 28^, Steve Shea (Brigham and Women's Hospital, Harvard University)^28^, Stephen Sidney (Kaiser Permanente Division of Research, Oakland, CA)^25^, Kevin Silverstein (University of Minnesota)^25^, David S. Siscovick (University of Washington)^5, 1, 25^, Nicholas L. Smith (University of Washington)^2, 15, 20, 23^, Nona Sotoodehnia (University of Washington)^3, 15, 23^, Asoke Srinivasan (Tougaloo College)^29^, Herman A. Taylor (Jackson State University, Tougaloo College, University of Mississippi Medical Center)^5,^ ^29^, Kent Taylor (Cedars-Sinai Medical Center)^31^, Fridtjof Thomas (University of Texas Health Science Center Houston)^3, 22^, Russell P. Tracy (University of Vermont)^5, 9, 11, 12, 14, 15, 17, 20, 23^, Michael Y. Tsai (University of Minnesota)^9, 31^, Kelly A. Volcik (University of Texas Health Science Center Houston)^22^, Chrstina L Wassel (University of California San Diego)^9, 15, 31^, Karol Watson (University of California Los Angeles)^31^, Gina Wei (National Heart, Lung, and Blood Institute)^25^, Wendy White (Tougaloo College)^29^, Kerri L. Wiggins (University of Vermont)^23^, Jemma B. Wilk (Boston University)^10, 28^, O. Dale Williams (Florida International University)^25^, Gregory Wilson (Jackson State University)^29^, James G. Wilson (University of Mississippi Medical Center)^1, 2, 5, 8, 9, 11, 12, 14, 17, 20, 29^, Phillip Wolf (Boston University)^28^, Neil A. Zakai (University of Vermont)^2, 23^

**ISGS and SWISS**

John Hardy (Reta Lila Weston Research Laboratories, Institute of Neurology, University College London)^18^, James F. Meschia (Mayo Clinic)^18^, Michael Nalls (National Institute on Aging)^2, 18^, Stephen S. Rich (University of Virginia)^2, 4, 7, 8, 9, 11, 14, 15, 17, 18, 31^, Andrew Singleton (National Institute on Aging)^18^, Brad Worrall (University of Virginia)^18^

**LungGO**

Michael J. Bamshad (Seattle Children's Hospital, University of Washington)^4, 6, 7, 8, 10, 11, 13, 15, 17, 27^, Kathleen C. Barnes (Johns Hopkins University)^2, 10, 14, 12, 15, 17, 20, 24, 30, 32^, Ibrahim Abdulhamid (Children’s Hospital of Michigan)^27^, Frank Accurso (University of Colorado)^27^, Ran Anbar (Upstate Medical University)^27^, Terri Beaty (Johns Hopkins University)^24, 30^, Abigail Bigham (University of Washington)^13, 15, 27^, Phillip Black (Children’s Mercy Hospital)^27^, Eugene Bleecker (Wake Forest University)^33^, Kati Buckingham (University of Washington)^27^, Anne Marie Cairns (Maine Medical Center)^27^, Wei-Min Chen (University of Virginia)^13, 15, 18^, Daniel Caplan (Emory University)^27^, Barbara Chatfield (University of Utah)^27^, Aaron Chidekel (A.I. Dupont Institute Medical Center)^27^, Michael Cho (Brigham and Women's Hospital, Harvard Medical School)^13, 15, 24^, David C. Christiani (Massachusetts General Hospital)^21^, James D. Crapo (National Jewish Health)^24, 30^, Julia Crouch (Seattle Children's Hospital)6, Denise Daley (University of British Columbia)^30^, Anthony Dang (University of North Carolina Chapel Hill)^26^, Hong Dang (University of North Carolina Chapel Hill)^26^, Alicia De Paula (Ochsner Health System)^27^, Joan DeCelie-Germana (Schneider Children’s Hospital)^27^, Allen Dozor (New York Medical College, Westchester Medical Center)^27,^ Mitch Drumm (University of North Carolina Chapel Hill)^26^, Maynard Dyson (Cook Children’s Med. Center)^27^, Julia Emerson (Seattle Children's Hospital, University of Washington)^27^, Mary J. Emond (University of Washington)^10, 13, 15, 17, 27^, Thomas Ferkol (St. Louis Children's Hospital, Washington University School of Medicine)^27^, Robert Fink (Children’s Medical Center of Dayton)^27^, Cassandra Foster (Johns Hopkins University)^30^, Deborah Froh (University of Virginia)^27^, Li Gao (Johns Hopkins University)^24, 30, 32^, William Gershan (Children’s Hospital of Wisconsin)^27^, Ronald L. Gibson (Seattle Children's Hospital, University of Washington)^10, 27^, Elizabeth Godwin (University of North Carolina Chapel Hill)^26^, Magdalen Gondor (All Children’s Hospital Cystic Fibrosis Center)^27^, Hector Gutierrez (University of Alabama at Birmingham)^27^, Nadia N. Hansel (Johns Hopkins University, Johns Hopkins University School of Public Health)^10, 15, 30^, Paul M. Hassoun (Johns Hopkins University)^10, 14, 32^, Peter Hiatt (Texas Children's Hospital)^27^, John E. Hokanson (University of Colorado)^24^, Michelle Howenstine (Indiana University, Riley Hospital for Children)^27^, Laura K. Hummer (Johns Hopkins University)^32^, Jamshed Kanga (University of Kentucky)^27^, Yoonhee Kim (National Human Genome Research Institute)^24, 32^, Michael R. Knowles (University of North Carolina Chapel Hill)^10, 26^, Michael Konstan (Rainbow Babies & Children’s Hospital)^27^, Thomas Lahiri (Vermont Children’s Hospital at Fletcher Allen Health Care)^27^, Nan Laird (Harvard School of Public Health)^24^, Christoph Lange (Harvard School of Public Health)^24^, Lin Lin (Harvard Medical School)^21^, Xihong Lin (Harvard School of Public Health)^21^, Tin L. Louie (University of Washington)^13, 15, 27^, David Lynch (National Jewish Health)^24^, Barry Make (National Jewish Health)^24^, Thomas R. Martin (University of Washington, VA Puget Sound Medical Center)^10, 21^, Steve C. Mathai (Johns Hopkins University)^32^, Rasika A. Mathias (Johns Hopkins University)^10, 13, 15, 30, 32^, John McNamara (Children’s Hospitals and Clinics of Minnesota)^27^, Sharon McNamara (Seattle Children's Hospital)^27^, Deborah Meyers (Wake Forest University)^33^, Susan Millard (DeVos Children’s Butterworth Hospital, Spectrum Health Systems)^27^, Peter Mogayzel (Johns Hopkins University)^27^, Richard Moss (Stanford University)^27^, Tanda Murray (Johns Hopkins University)^30^, Dennis Nielson (University of California at San Francisco)^27^, Blakeslee Noyes (Cardinal Glennon Children’s Hospital)^27^, Wanda O'Neal (University of North Carolina Chapel Hill)^26^, David Orenstein (Children’s Hospital of Pittsburgh)^27^, Brian O'Sullivan (University of Massachusetts Memorial Health Care)^27^, Rhonda Pace (University of North Carolina Chapel Hill)^26^, Peter Pare (St. Paul’s Hospital)^30^, H. Worth Parker (Dartmouth-Hitchcock Medical Center, New Hampshire Cystic Fibrosis Center)^27^, Mary Ann Passero (Rhode Island Hospital)^27^, Elizabeth Perkett (Vanderbilt University)^27,^ Adrienne Prestridge (Children's Memorial Hospital)^27^, Nicholas M. Rafaels (Johns Hopkins University)^30^, Bonnie Ramsey (Seattle Children's Hospital, University of Washington)^27^, Elizabeth Regan (National Jewish Health)^24^, Clement Ren (University of Rochester)^27^, George Retsch-Bogart (University of North Carolina Chapel Hill)^27^, Michael Rock (University of Wisconsin Hospital and Clinics)^27^, Antony Rosen (Johns Hopkins University)^32^, Margaret Rosenfeld (Seattle Children's Hospital, University of Washington)^27^, Ingo Ruczinski (Johns Hopkins University School of Public Health)^13, 15, 30^, Andrew Sanford (University of British Columbia)^30^, David Schaeffer (Nemours Biomedical Research Program is a funding contributor to the EPIC Observational Study)^27^, Cindy Sell (University of North Carolina Chapel Hill)^26^, Daniel Sheehan (Children's Hospital of Buffalo)^27^, Edwin K. Silverman (Brigham and Women's Hospital, Harvard Medical School)^24, 30^, Don Sin (Children’s Medical Center of Dayton)^30^, Terry Spencer (Elliot Health System)^27^, Jackie Stonebraker (University of North Carolina Chapel Hill)^26^, Holly K. Tabor (Seattle Children's Hospital, University of Washington)^6, 10, 11, 17, 27^, Laurie Varlotta (St. Christopher’s Hospital for Children)^27^, Candelaria I. Vergara (Johns Hopkins University)^30^, Robert Weiss ^30^, Fred Wigley (Johns Hopkins University)^32^, Robert A. Wise (Johns Hopkins University)^30^, Fred A. Wright (University of North Carolina Chapel Hill)^26^, Mark M. Wurfel (University of Washington)^10, 14, 21^, Robert Zanni (Monmouth Medical Center)^27^, Fei Zou (University of North Carolina Chapel Hill)^26^

**SeattleGO**

Deborah A. Nickerson (University of Washington)^3, 4, 5, 7, 8, 9, 11, 15, 17, 18, 19^, Mark J. Rieder (University of Washington)^4, 11, 13, 15, 16, 17, 19^, Phil Green (University of Washington), Jay Shendure (University of Washington)^1, 8, 14, 16, 17^, Joshua M. Akey (University of Washington)^13, 14, 15^, Michael J. Bamshad (Seattle Children's Hospital, University of Washington)^4, 6, 7, 8, 10, 11, 13, 15, 17, 27^, Carlos D. Bustamante (Stanford University School of Medicine)^3, 13, 15^, David R. Crosslin (University of Washington)^2, 9^, Evan E. Eichler (University of Washington)^19^, P. Keolu Fox^2^, Wenqing Fu (University of Washington)^13^, Adam Gordon (University of Washington)^11^, Simon Gravel (Stanford University School of Medicine)^13, 15^, Gail P. Jarvik (University of Washington)^9, 15^, Jill M. Johnsen (Puget Sound Blood Center, University of Washington)^2^, Mengyuan Kan (Baylor College of Medicine)^13^, Eimear E. Kenny (Stanford University School of Medicine)^3, 13, 15^, Jeffrey M. Kidd (Stanford University School of Medicine)^13, 15^, Fremiet Lara-Garduno (Baylor College of Medicine)^15^, Suzanne M. Leal (Baylor College of Medicine)^1, 13, 15, 16, 17, 19, 20^, Dajiang J. Liu (Baylor College of Medicine)^13, 15^, Sean McGee (University of Washington)^13, 15, 19^, Timothy D. O’Connor (University of Washington)^13^, Bryan Paeper (University of Washington)^16^, Peggy D. Robertson (University of Washington)^4^, Joshua D. Smith (University of Washington)^4, 16, 19^, Jeffrey C. Staples (University of Washington), Jacob A. Tennessen (University of Washington)^13^, Emily H. Turner (University of Washington)^4, 16^, Gao Wang (Baylor College of Medicine)^1,13,20^, Qian Yi (University of Washington)^4^

**WHISP**

Rebecca Jackson (Ohio State University)^1, 2, 4, 5, 8, 12, 14, 15, 17, 18, 20, 34^, Kari North (University of North Carolina Chapel Hill)^1, 3, 9, 10, 13, 15, 17, 34,^ Ulrike Peters (Fred Hutchinson Cancer Research Center)^1, 3, 11, 12, 13, 15, 17, 18, 34^, Christopher S. Carlson (Fred Hutchinson Cancer Research Center, University of Washington)^1, 2, 3, 5, 12, 13, 14, 15, 16, 17, 18, 19, 34^, Garnet Anderson (Fred Hutchinson Cancer Research Center)^34^, Hoda Anton-Culver (University of California at Irvine)^34^, Themistocles L. Assimes (Stanford University School of Medicine)^5, 9, 11, 34^, Paul L. Auer (Fred Hutchinson Cancer Research Center)^1, 2, 3^, ^5, 11, 12, 13, 15, 16, 18, 34^, Shirley Beresford (Fred Hutchinson Cancer Research Center)^34^, Chris Bizon (University of North Carolina Chapel Hill)^3, 9, 13, 15, 34^, Henry Black (Rush Medical Center)^34^, Robert Brunner (University of Nevada)^34^, Robert Brzyski (University of Texas Health Science Center San Antonio)^34^, Dale Burwen (National Heart, Lung, and Blood Institute WHI Project Office)^34^, Bette Caan (Kaiser Permanente Division of Research, Oakland, CA)^34^, Cara L. Carty (Fred Hutchinson Cancer Research Center)^18, 34^, Rowan Chlebowski (Los Angeles Biomedical Research Institute)^34^, Steven Cummings (University of California at San Francisco)^34^, J. David Curb* (University of Hawaii)^9, 18, 34^, Charles B. Eaton (Brown University, Memorial Hospital of Rhode Island)^12, 34^, Leslie Ford (National Heart, Lung, and Blood Institute, National Heart, Lung, and Blood Institute WHI Project Office)^34^, Nora Franceschini (University of North Carolina Chapel Hill)^2, 3, 9, 10, 15, 34^, Stephanie M. Fullerton (University of Washington)^6, 11, 34^, Margery Gass (University of Cincinnati)^34^, Nancy Geller (National Heart, Lung, and Blood Institute WHI Project Office)^34^, Gerardo Heiss (University of North Carolina Chapel Hill)^5, 34^, Barbara V. Howard (Howard University, MedStar Research Institute)34, Li Hsu (Fred Hutchinson Cancer Research Center)^1, 13, 15, 18, 34^, Carolyn M. Hutter (Fred Hutchinson Cancer Research Center)^13, 15, 18, 34^, John Ioannidis (Stanford University School of Medicine)^11, 34^, Shuo Jiao (Fred Hutchinson Cancer Research Center)^34^, Karen C. Johnson (University of Tennessee Health Science Center)^3, 34^, Charles Kooperberg (Fred Hutchinson Cancer Research Center)^1, 5, 9, 13, 14, 15, 17, 18, 34^, Lewis Kuller (University of Pittsburgh)^34^, Andrea LaCroix (Fred Hutchinson Cancer Research Center)^34^, Kamakshi Lakshminarayan (University of Minnesota)^18, 34^, Dorothy Lane (State University of New York at Stony Brook)^34^, Ethan M. Lange (University of North Carolina Chapel Hill)^3, 5, 9, 13, 34^, Leslie A. Lange (University of North Carolina Chapel Hill)^1, 2, 3, 5, 9, 12, 13, 15, 17, 18, 20, 25, 34^, Norman Lasser (University of Medicine and Dentistry of New Jersey)^34^, Erin LeBlanc (Kaiser Permanente Center for Health Research, Portland, OR)^34^, Cora E. Lewis (University of Alabama at Birmingham)^25,34^, Kuo-Ping Li (University of North Carolina Chapel Hill)^9, 34^, Marian Limacher (University of Florida)^34^, Dan-Yu Lin (University of North Carolina Chapel Hill)^1, 3, 9, 13, 15, 34^, Benjamin A. Logsdon (Fred Hutchinson Cancer Research Center)^2, 34^, Shari Ludlam (National Heart, Lung, and Blood Institute WHI Project Office)^34^, JoAnn E. Manson (Brigham and Women's Hospital, Harvard School of Public Health)^34^, Karen Margolis (University of Minnesota)^34^, Lisa Martin (George Washington University Medical Center)^9, 34^, Joan McGowan (National Heart, Lung, and Blood Institute WHI Project Office)^34^, Keri L. Monda (Amgen, Inc.)^1,^ ^15, 34^, Jane Morley Kotchen (Medical College of Wisconsin)^34^, Lauren Nathan (University of California Los Angeles)^34^, Judith Ockene (Fallon Clinic, University of Massachusetts)^34^, Mary Jo O'Sullivan (University of Miami)^34^, Lawrence S. Phillips (Emory University)^34^, Ross L. Prentice (Fred Hutchinson Cancer Research Center)^34^, Alexander P. Reiner (Fred Hutchinson Cancer Research Center, University of Washington)^1, 2, 3, 5, 9, 11, 12, 13, 14, 15, 20, 25, 34^, John Robbins (University of California at Davis)^34^, Jennifer G. Robinson (University of Iowa)^9, 11, 18, 34^, Jacques E. Rossouw (National Heart, Lung, and Blood Institute, National Heart, Lung, and Blood Institute WHI Project Office)^5, 14, 17, 20, 34^, Haleh Sangi-Haghpeykar (Baylor College of Medicine)^34^, Gloria E. Sarto (University of Wisconsin)^34^, Sally Shumaker (Wake Forest University)^34^, Michael S. Simon (Wayne State University)^34^, Marcia L. Stefanick (Stanford University School of Medicine)^34^, Evan Stein (Medical Research Labs)^34^, Hua Tang (Stanford University)^2, 34^, Kira C. Taylor (University of Louisville)^1, 3, 13, 15, 20, 34^, Cynthia A. Thomson (University of Arizona)^34^, Timothy A. Thornton (University of Washington)^13, 15, 18, 34^, Linda Van Horn (Northwestern University)^34^, Mara Vitolins (Wake Forest University)^34^, Jean Wactawski-Wende (University of Buffalo)^34^, Robert Wallace (University of Iowa)^2, 34^, Sylvia Wassertheil-Smoller (Boston University)^18, 34^, Donglin Zeng (University of North Carolina Chapel Hill)^9, 34^

*deceased

**NHLBI GO ESP Project Team**

Deborah Applebaum-Bowden (National Heart, Lung, and Blood Institute)^4, 7, 12, 17^, Michael Feolo (National Center for Biotechnology Information)^12^, Weiniu Gan (National Heart, Lung, and Blood Institute)^7, 8, 16, 17^, Dina N. Paltoo (National Heart, Lung, and Blood Institute)^4, 6, 11, 17^, Jacques E. Rossouw (National Heart, Lung, and Blood Institute, National Heart, Lung, and Blood Institute WHI Project Office)^5, 14, 17, 20, 34^, Phyliss Sholinsky (National Heart, Lung, and Blood Institute)^4, 12, 17^, Anne Sturcke (National Center for Biotechnology Information)^12^

**ESP Groups**

^1^Anthropometry Project Team, ^2^Blood Count/Hematology Project Team, ^3^Blood Pressure Project Team, ^4^Data Flow Working Group, ^5^Early MI Project Team, ^6^ELSI Working Group, ^7^Executive Committee, ^8^Family Study Project Team, ^9^Lipids Project Team, ^10^Lung Project Team, ^11^Personal Genomics Project Team, ^12^Phenotype and Harmonization Working Group, ^13^Population Genetics and Statistical Analysis Working Group, ^14^Publications and Presentations Working Group, ^15^Quantitative Analysis Ad Hoc Task Group, ^16^Sequencing and Genotyping Working Group, ^17^Steering Committee, ^18^Stroke Project Team, ^19^Structural Variation Working Group, ^20^Subclinical/Quantitative Project Team

**ESP Cohorts**

^21^Acute Lung Injury (ALI), ^22^Atherosclerosis Risk in Communities (ARIC), ^23^Cardiovascular Health Study (CHS), ^24^Chronic Obstructive Pulmonary Disease (COPDGene), ^25^Coronary Artery Risk Development in Young Adults (CARDIA), ^26^Cystic Fibrosis (CF), ^27^Early Pseudomonas Infection Control (EPIC), ^28^Framingham Heart Study (FHS), ^29^Jackson Heart Study (JHS), ^30^Lung Health Study (LHS), ^31^Multi-Ethnic Study of Atherosclerosis (MESA), ^32^Pulmonary Arterial Hypertension (PAH), ^33^Severe Asthma Research Program (SARP), ^34^Women's Health Initiative (WHI)
